# Supplementary figures and images for: Unique trackway on Permian Karoo shoreline provides evidence of temnospondyl locomotory behaviour
Source: PLoS One. 2023 Mar 29;18(3):e0282354. doi: 10.1371/journal.pone.0282354 (PMC10057796; doi:10.1371/journal.pone.0282354)

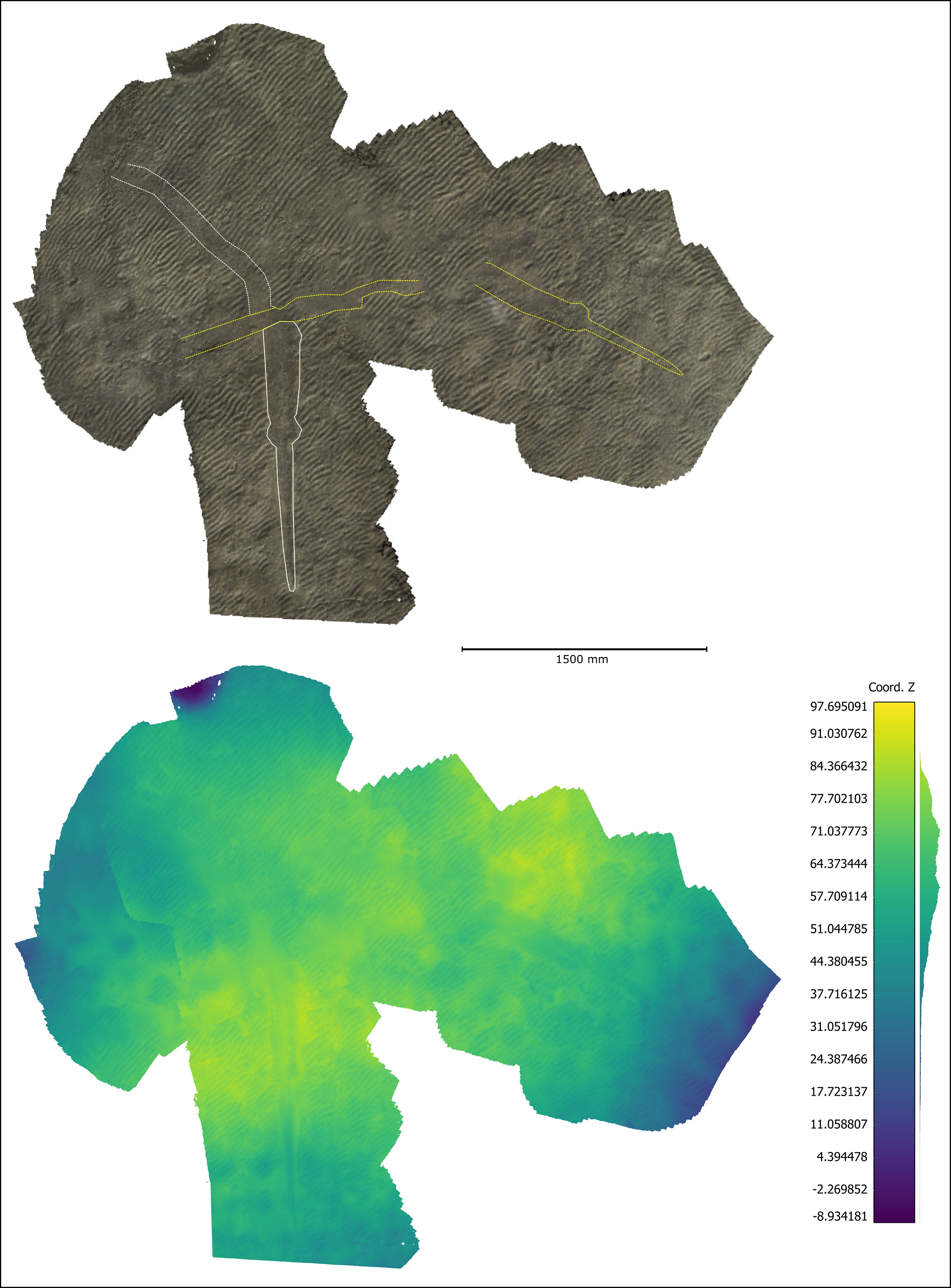

Supplement: S1 Fig — Textured scan (top), with the outlines of the impressions and associated swim trails of impression 3 (White) and impression 7 (Yellow), shown next to the false-colour depth model (bottom). Depth scale is in mm. (TIF) [file pone.0282354.s001.tif]

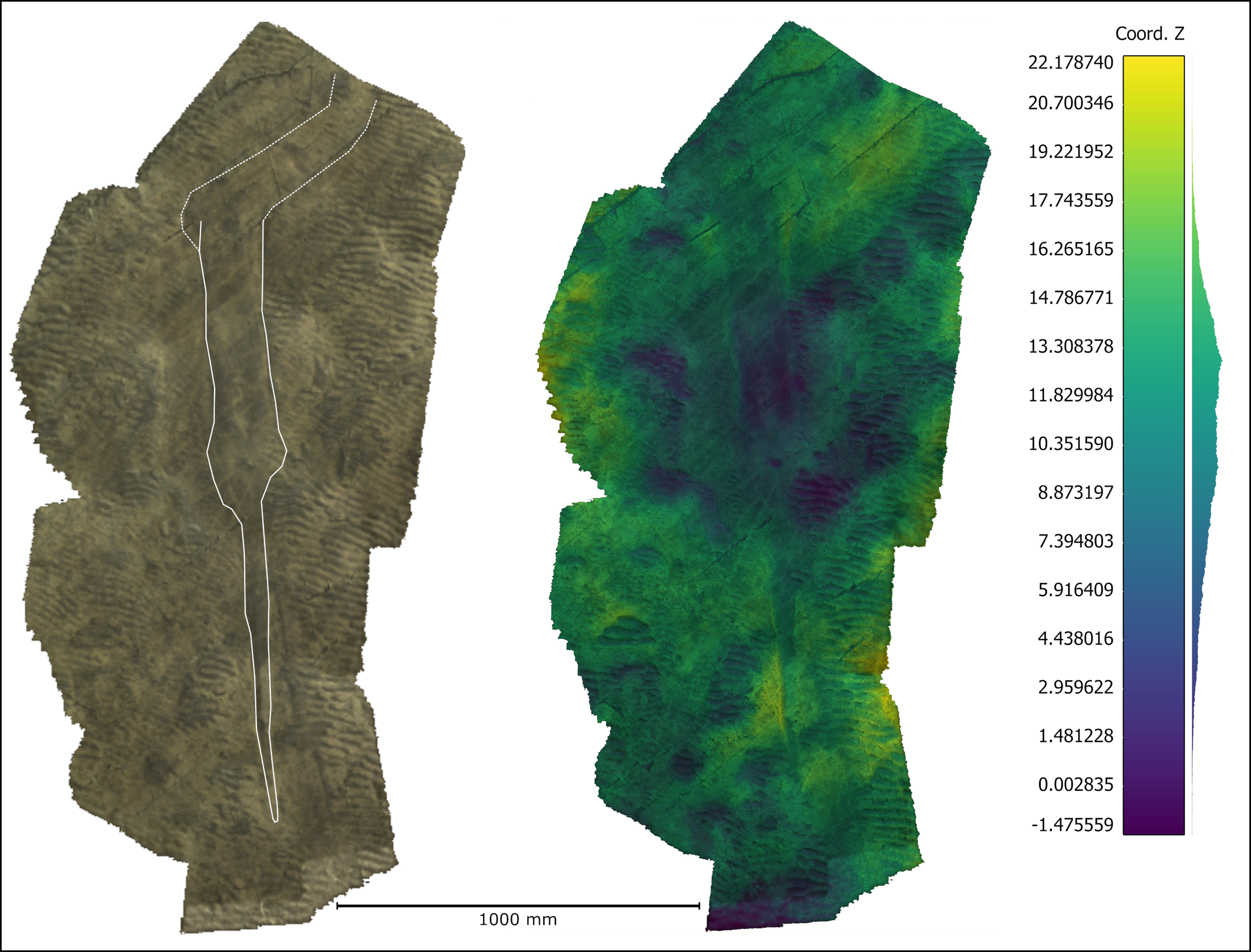

Supplement: S2 Fig — Textured scan (Left) with the outline of the impression and swim trace shown next to the false-colour depth model (Right). Depth scale is in mm. (TIF) [file pone.0282354.s002.tif]

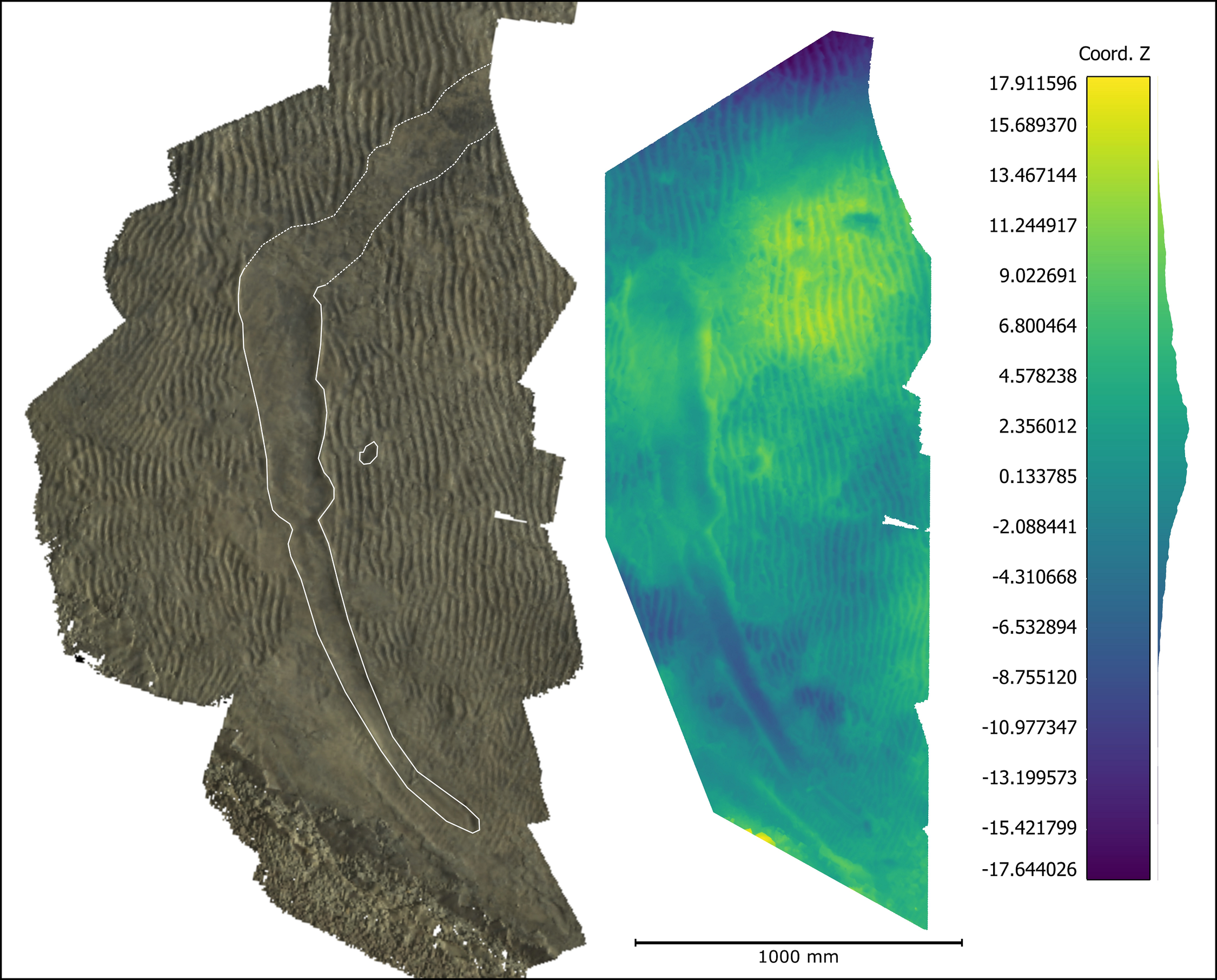

Supplement: S3 Fig — Textured scan (Left) with the outline of the impression and swim trace shown next to the false-colour depth model (Right). Depth scale is in mm. (TIF) [file pone.0282354.s003.tif]
