# Supplementary material for: Unique trackway on Permian Karoo shoreline provides evidence of temnospondyl locomotory behaviour
Source: PLoS One. 2023 Mar 29;18(3):e0282354. doi: 10.1371/journal.pone.0282354 (PMC10057796; doi:10.1371/journal.pone.0282354)
Supplement: Attachment — Submitted filename: PONE-D-22-30091_reviewerEM.pdf [file pone.0282354.s005.pdf]

PLOS ONE

Unique trackway on Permian Karoo shoreline provides evidence of temnospondyl locomotory behaviour.

--Manuscript Draft--

Manuscript Number:	PONE-D-22-30091
Article Type:	Research Article
Full Title:	Unique trackway on Permian Karoo shoreline provides evidence of temnospondyl locomotory behaviour.
Short Title:	A late Permian shallow water palaeosurface from KwaZulu-Natal.
Corresponding Author:	David Patrick Groenewald, Ph.D.<br>University of the Witwatersrand Johannesburg<br>Braamfontein, Gauteng SOUTH AFRICA
Keywords:	Ichnofossils; Ecca Group; Beaufort Group; Waterford Formation; Karoo Basin; Ecca-Beaufort contact; Rhinesuchidae; Temnospondyl; tracefossils; locomotion; palaeosurface
Abstract:	Large-bodied temnospondyl amphibians were the dominant predators in non-marine aquatic ecosystems from the Carboniferous to the Middle Triassic. In the Permian-aged lower Beaufort Group of the main Karoo Basin, South Africa, temnospondyls are represented exclusively by the family Rhinesuchidae and are well represented by body fossils, whereas trace fossils are scarce. Accordingly, most interpretations of the behaviour of this family are based on skeletal morphology and histological data. Here we document the sedimentology and palaeontology of a late Permian palaeosurface situated immediately below the paleaoshoreline of the Ecca Sea (transition from the Ecca Group to the Beaufort Group) near the town of Estcourt in KwaZulu-Natal Province. The surface preserves numerous ichnofossils, including tetrapod footprints and fish swim-trails, but most striking are seven body impressions and associated swim trails that we attribute to a medium-sized (~1.9 m long) rhinesuchid temnospondyl. These provide valuable insight into the behaviour of these animals. Sinuous marks within some of the traces suggest that the trackmaker swam with continuous sub-undulatory propulsion of the tail.
Order of Authors:	David Patrick Groenewald, Ph.D.
	Ashley Krüger, Ph.D
	Michael O. Day, Ph.D
	Cameron R. Penn-Clarke, Ph.D
	P. John Hancox, Ph.D
	Bruce S. Rubidge, Ph.D
Additional Information:	
Question	Response
**Financial Disclosure**<br><br>Enter a financial disclosure statement that describes the sources of funding for the work included in this submission. Review the submission guidelines for detailed requirements. View published research articles from *PLOS ONE* for specific examples.<br><br>This statement is required for submission	The financial support of GENUS [DSI-NRF Centre of Excellence in Palaeosciences (CoE-Pal)], the National Research Foundation (NRF) and its African Origins Platform, and the Palaeontological Scientific Trust (PAST) and its Scatterlings of Africa programmes towards this research is hereby acknowledged.The funders had no role in study design, data collection and analysis, decision to publish, or preparation of the manuscript.

Powered by Editorial Manager® and ProduXion Manager® from Aries Systems Corporation

and **will appear in the published article** if the submission is accepted. Please make sure it is accurate.<br><br>**Unfunded studies**<br>Enter: *The author(s) received no specific funding for this work.*<br><br>**Funded studies**<br>Enter a statement with the following details:<br>• Initials of the authors who received each award<br>• Grant numbers awarded to each author<br>• The full name of each funder<br>• URL of each funder website<br>• Did the sponsors or funders play any role in the study design, data collection and analysis, decision to publish, or preparation of the manuscript?<br>• **NO** - Include this sentence at the end of your statement: *The funders had no role in study design, data collection and analysis, decision to publish, or preparation of the manuscript.*<br>• **YES** - Specify the role(s) played.<br><br>* typeset	
**Competing Interests**<br><br>Use the instructions below to enter a competing interest statement for this submission. On behalf of all authors, disclose any competing interests that could be perceived to bias this work—acknowledging all financial support and any other relevant financial or non-financial competing interests.<br><br>This statement is required for submission and **will appear in the published article** if the submission is accepted. Please make sure it is accurate and that any funding sources listed in your Funding Information later in the submission form are also declared in your Financial Disclosure statement.<br><br>View published research articles from *PLOS ONE* for specific examples.	The authors have declared that no competing interests exist.

**NO authors have competing interests**<br><br>Enter: *The authors have declared that no competing interests exist.*<br><br>**Authors with competing interests**<br><br>Enter competing interest details beginning with this statement:<br><br>*I have read the journal's policy and the authors of this manuscript have the following competing interests: [insert competing interests here]*<br><br>* typeset	
**Ethics Statement**<br><br>Enter an ethics statement for this submission. This statement is required if the study involved:<br><br>• Human participants<br>• Human specimens or tissue<br>• Vertebrate animals or cephalopods<br>• Vertebrate embryos or tissues<br>• Field research<br><br>Write "N/A" if the submission does not require an ethics statement.<br><br>General guidance is provided below. Consult the submission guidelines for detailed instructions. **Make sure that all information entered here is included in the Methods section of the manuscript.**	N/A

**Format for specific study types**<br><br>**Human Subject Research (involving human participants and/or tissue)**<br>• Give the name of the institutional review board or ethics committee that approved the study<br>• Include the approval number and/or a statement indicating approval of this research<br>• Indicate the form of consent obtained (written/oral) or the reason that consent was not obtained (e.g. the data were analyzed anonymously)<br><br>**Animal Research (involving vertebrate animals, embryos or tissues)**<br>• Provide the name of the Institutional Animal Care and Use Committee (IACUC) or other relevant ethics board that reviewed the study protocol, and indicate whether they approved this research or granted a formal waiver of ethical approval<br>• Include an approval number if one was obtained<br>• If the study involved *non-human primates*, add *additional details* about animal welfare and steps taken to ameliorate suffering<br>• If anesthesia, euthanasia, or any kind of animal sacrifice is part of the study, include briefly which substances and/or methods were applied<br><br>**Field Research**<br><br>Include the following details if this study involves the collection of plant, animal, or other materials from a natural setting:<br>• Field permit number<br>• Name of the institution or relevant body that granted permission	
**Data Availability**<br><br>Authors are required to make all data underlying the findings described fully available, without restriction, and from the time of publication. PLOS allows rare exceptions to address legal and ethical concerns. See the PLOS Data Policy and FAQ for detailed information.	Yes - all data are fully available without restriction

Powered by Editorial Manager® and ProduXion Manager® from Aries Systems Corporation

A Data Availability Statement describing where the data can be found is required at submission. Your answers to this question constitute the Data Availability Statement and **will be published in the article**, if accepted.<br><br>**Important:** Stating 'data available on request from the author' is not sufficient. If your data are only available upon request, select 'No' for the first question and explain your exceptional situation in the text box.<br><br>Do the authors confirm that all data underlying the findings described in their manuscript are fully available without restriction?	
**Describe where the data may be found in full sentences. If you are copying our sample text, replace any instances of XXX with the appropriate details.**<br><br>• If the data are **held or will be held in a public repository**, include URLs, accession numbers or DOIs. If this information will only be available after acceptance, indicate this by ticking the box below. For example: *All XXX files are available from the XXX database (accession number(s) XXX, XXX.)*.<br>• If the data are all contained **within the manuscript and/or Supporting Information files**, enter the following: *All relevant data are within the manuscript and its Supporting Information files.*<br>• If neither of these applies but you are able to provide **details of access elsewhere**, with or without limitations, please do so. For example:<br><br>*Data cannot be shared publicly because of [XXX]. Data are available from the XXX Institutional Data Access / Ethics Committee (contact via XXX) for researchers who meet the criteria for access to confidential data.*<br><br>*The data underlying the results presented in the study are available from (include the name of the third party*	All relevant data are within the manuscript and its Supporting Information files.

and contact information or URL).<br>• This text is appropriate if the data are owned by a third party and authors do not have permission to share the data.<br><br>* typeset	
Additional data availability information:	

Manuscript

Click here to access/download;Manuscript;Groenewald et al. Amphibian trackway.docx

Unique trackway on Permian Karoo shoreline provides evidence of temnospondyl locomotory behaviour.

Short Title: A late Permian shallow water palaeosurface from KwaZulu-Natal.

Groenewald, D.P.[1,2*], Krüger, A.[3], Day, M.O.[4,1], Penn-Clarke, C.R.[5,1], Hancox, P.J.[1], Rubidge, B.S.[1]

Large-bodied temnospondyl amphibians were the dominant predators in non-marine aquatic ecosystems from the Carboniferous to the Middle Triassic. In the Permian-aged lower Beaufort Group of the main Karoo Basin, South Africa, temnospondyls are represented exclusively by the family Rhinesuchidae and are well represented by body fossils, whereas trace fossils are scarce. Accordingly, most interpretations of the behaviour of this family are based on skeletal morphology and histological data. Here we document the sedimentology and palaeontology of a late Permian palaeosurface situated immediately below the paleaoshoreline of the Ecca Sea (transition from the Ecca Group to the Beaufort Group) near the town of Estcourt in KwaZulu-Natal Province. The surface preserves numerous ichnofossils, including tetrapod footprints and fish swim-trails, but most striking are seven body impressions and associated swim trails that we attribute to a medium-sized (~1.9 m long) rhinesuchid temnospondyl. These provide valuable insight into the behaviour of these animals. Sinuous marks within some of the traces suggest that the trackmaker swam with continuous sub-undulatory propulsion of the tail.

Keywords: Ichnofossils; Ecca Group; Beaufort Group; Waterford Formation; Karoo Basin; Ecca-Beaufort contact; Rhinesuchid

Authors:

[1]Evolutionary Studies Institute, University of the Witwatersrand, Johannesburg, South Africa

[2]Institut Català de Paleontologia Miquel Crusafont, ICTA-ICP building, c/ de les columnes, s/n, E-08193 Cerdanyola del Vallès, Spain

[3]Department of Palaeobiology, Swedish Royal Museum of Natural History, Stockholm, Sweden

[4]Natural History Museum, London, United Kingdom

[5]Council for Geoscience, Bellville, Cape Town, South Africa

*Corresponding author:

E-mail: david.groenewald@wits.ac.za (DPG)

1 INTRODUCTION

The main Karoo Basin is renowned for its rich vertebrate body fossil record, as well as its extensive ichnological record comprising terrestrial and aquatic traces from the Upper Pennsylvanian to the Lower Jurassic. A diverse array of trace fossils has been recognised with attributed trace- and trackmakers including fish (e.g. Anderson 1976; Sciscio *et al.* 2020), invertebrates (e.g. Anderson 1981; Bordy *et al.* 2011; Gastaldo & Rolerson 2008; Hobday & Tavener-Smith 1975; Mason & Christie 1986; Savage 1971; Smith 1993; Stanistreet *et al.* 1980), therapsids (Bordy *et al.* 2019; de Klerk 2002; MacRae 1990; Marchetti *et al.* 2019, 2020; Porchetti & Nicosia 2007; Smith 1993), dinosaurs and reptiles (Bordy 2021; Bordy *et al.* 2020; Ellenberger 1970, 1972, 1974; Olsen & Galton 1984; Porchetti & Nicosia 2007; Raath *et al.* 1990; Raath & Yates 2005; Sciscio *et al.* 2016; Van Dijk 1978; Wilson *et al.* 2009), and amphibians (Cisneros *et al.* 2020; Ellenberger 1974; Marsicano *et al.* 2014; Van Dijk *et al.* 2002).

A large palaeosurface with several remarkably well-preserved trace fossils is exposed along the course of a tributary of the Rensburgspruit in the Estcourt district, KwaZulu-Natal Province. The most striking ichnofossils are of seven large (>1 m long) impressions preserving resting and locomotor behaviours. While the unique morphology of these traces and the importance of the site has previously been recognised (e.g. Rossouw 2010), neither the site nor the trackways have been fully described. This is mainly because the size (> 1 m) and shallow depth (< 5 mm) of the impressions resulted in traditional casting methods being unsuccessful. Other traces preserved on the palaeosurface include numerous smaller (10 – 15 cm diameter) circular depressions and paired and unpaired linear traces.

In this paper, we document the site with its sedimentary structures and unique traces as well as its stratigraphic context. Using high-resolution three-dimensional (3D) photogrammetry scans and aerial orthophotographs, we provide the first comprehensive description of the

large impressions. A probable trackmaker for the impressions is assigned and locomotory behaviour of the trackmaker is inferred.

2 GEOLOGICAL AND PALAEONTOLOGICAL CONTEXT

2.1 Geological background and stratigraphy of the palaeosurface

Named in honour of the late Mr Dave Green, who discovered this remarkable site and had a passion for palaeontology, the Dave Green palaeosurface is located along a tributary of the Rensburgspruit on the farm Van der Merwe’s Kraal 972, approximately 10 km northeast of the town Estcourt in KwaZulu-Natal Province, South Africa (Figs. 1 and 2). The region spans the Ecca-Beaufort transition and is primarily underlain by ~~late~~ Permian (Changhsingian) siliciclastic deposits of the Waterford and Balfour formations that are intruded by lower Jurassic dolerite dykes and sills (Council for Geoscience 1998; Geological Survey 1981; Green 1997; Groenewald 2015, 2021; Groenewald *et al.* 2022). The Dave Green palaeosurface covers an area of approximately 600 m^2 and is situated in the upper Waterford Formation, immediately below the Ecca-Beaufort contact recording the transition from marine/ lacustrine to fluvial environmental conditions (Green 1997; Groenewald 2015, 2021; Groenewald *et al.* 2022; Rossouw 2010).

Fig. 1. Geological setting of the Dave Green palaeosurface. A) Simplified geological map of the main Karoo Basin. Position of the study area is indicated. B) Aerial view of the palaeosurface. C) Stratigraphic log measured along the Rensburgspruit.

Fig. 2. Orthophoto of the palaeosurface with the positions of the seven large impressions and paths followed for the high-resolution scans indicated. Numbers used for the impressions are the same throughout the ~~manuscript~~.

In the southern Karoo Basin, the Waterford Formation constitutes the uppermost Ecca Group (Johnson 1976; Johnson *et al.* 2006; Mason *et al.* 2015; Rubidge *et al.* 2000, 2012; SACS 1980), but has recently been shown to also be present in the north of the basin, where it gradationally overlies the Volksrust or Tierberg formations (Groenewald *et al.* 2022). This diagnostic and easily recognisable ~~F~~ormation, which incorporates strata previously assigned

to the upper Volksrust and/or lower Balfour (including the now defunct Normandien and Estcourt) formations (Groenewald 2021; Groenewald *et al.* 2022), is characterized as being generally arenaceous in nature, generally ripple-marked, as well as containing rhythmically-bedded carbonaceous shale and ubiquitous soft sediment deformation structures (Fig. 3).

Fig. 3. Features of the upper Waterford and lower Balfour formations in the study area. A) Large calcareous nodules. B) Rounded/ Smoothed symmetrical ripples on the top of the thick sandstone unit. C) Dark grey, laminated mudstones. D) Iron oxide haloes in sandstone unit. E) Soft sediment deformation. Note how the laminations follow the deformation bed. F) Sequence of rhythmites, comprising alternating mudstone and sandstone units. G) Large symmetrical ripples with sharp, straight crests and *Skolithos* burrows. H) Yellow-green mudstones typical of this facies association. I) Sandstone lens showing lateral/ downstream accretion. J) Calcareous concretions within a sandstone. K) Slickenside surfaces and nodules in mudstones. L) Fossilised wood weathering out of the mudstones. M) Horsetail impressions. N) *Glossopteris* leaf impressions. O) Large, unidentified bone c.f. dicynodont.

Previous studies ~~suggest~~ that these deposits accumulated in a regressive delta front and delta plain (Botha & Linström 1978; Green 1997; Groenewald *et al.* 2022; Linström 1987; Rubidge *et al.* 2000; SACS 1980; Smith *et al.* 1993). Mudstones of the Waterford Formation are commonly dark to medium grey (N3 – N5) and Olive Green (5GY 3/2), whereas siltstones and sandstones are typically lighter, varying between medium light grey to light grey (N6 – N8), and occasionally light brown (5YR 6/4). Laminated mudstones typically display platy/ flaky weathering and may contain fragmentary plant impressions and invertebrate traces.

The overlying Balfour Formation is, with respect to the Waterford Formation, markedly more argillaceous, with interspersed sandstone lenses that typically have an erosional base. In contrast to the dark grey to olive-green mudstones of the underlying Waterford Formation, mudstones of the Balfour Formation are generally moderate yellow (5Y 7/6) to dark greenish yellow (10Y 6/6), are massive to finely-laminated, display a blockier weathering pattern, and may contain calcareous nodules and slickensided surfaces (Fig. 3). Plant impressions and tetrapod vertebrate remains are more abundant and complete in the Balfour Formation than the underlying Waterford Formation. The depositional environment for the Balfour Formation in the northeastern main Karoo Basin has been interpreted as having accumulated

in high-load meandering river ~~environments~~ (Botha & Linström 1977, 1978; Green 1997; Groenewald 2021, 1989, 1990; Groenewald *et al.* 2022; Johnson *et al.* 2006; Muntingh 1989, 1997).The depositional environment for the Balfour Formation in the northeastern main Karoo Basin has been interpreted as high load meandering river environments (Botha & Linström 1977, 1978; Green 1997; Groenewald 2021, 1989, 1990; Groenewald *et al.* 2022; Johnson *et al.* 2006; Muntingh 1989, 1997).

2.2 Palaeontology

Van der Merwe's Kraal 972, as well its immediate surrounds, is palaeontologically rich, comprising a diverse assemblage of plant, insect, and vertebrate body fossils in addition to trace fossils (van Dijk 1997; van Dijk & Geertsema 2004; Geertsema *et al.* 2002; Green 1997; Lacey *et al.* 1975; Rossouw 2010; Selover & Gastaldo 2005). Green (1997) undertook detailed sedimentological and palaeontological work on the farm but although she noted and briefly discussed the Dave Green palaeosurface in her "shoreline log", she largely ignored the tetrapod traces. Green (1997) did, however, document 15 trackways from two surfaces roughly 1.1 km to the south of the Dave Green palaeosurface. These surfaces, which have since been re-covered by silt and mud, had been exposed during the excavation of a dam and Green (1997) considered them to be from a laterally equivalent stratigraphic level as the Dave Green Palaeosurface. The 15 trackways were attributed to small-to-medium sized dicynodont trackmakers and interpreted them as reflecting undertracks and movement of an animal buoyed by water (Green 1997). Plant fossils found near the Dave Green palaeosurface include impressions of *Glossopteris* leaves and sphenophyte stems (*Calmites* and *Paracalamites*; D.G. pers. obs.), as well as silicified wood identified as *Agathoxylon africanum* (M. Bamford pers. comm. 2019; Groenewald 2021) (Fig. 3). Vertebrate fossils recovered by us and previous workers from the farm van der Merwe's Kraal 972 include an almost complete rhinesuchid amphibian skull (BP/1/7858; c.f. *Laccosaurus*), fragmentary

dicynodont material, and fish scales. Although the body fossils are not diagnostic, the lowermost vertebrate assemblage zone of the Beaufort Group in this part of KZN is the late Permian *Daptocephalus* Assemblage Zone (AZ), although it is unclear which subzone is present (Groenewald *et al.* 2022; Viglietti 2020).

3 MATERIALS AND METHODS

We recorded the lithostratigraphy and sedimentology of the study area by measuring a stratigraphic section through the exposures of interbedded sandstones, siltstones and mudstones along the stream bed that crosses the palaeosurface. For this we used a Jacob's staff and Abney level, noting the lithology, colour, sedimentary structures, and fossils present. These data were used to characterise lithofacies and lithofacies associations as well as architectural elements after Miall (1996, 2016) to provide palaeoenvironmental context to the deposits (Table 1). A recent detailed appraisal of the sedimentology and lithostratigraphy across the Ecca-Beaufort contact in the north of the main Karoo Basin may be found in Groenewald *et al.* (2022) and is referred to herein.

Table 1. Descriptions and interpretations of lithofacies types [modified after Miall (2006); * denotes newly erected lithofacies types from Groenewald *et al.* (2022)] from the studied stratigraphic interval.

Lithofacies Code	Lithofacies	Description	Interpretation
St	Trough cross-bedded sandstone	Medium- to coarse-grained sandstones with cross-bedded units in which the lower bounding surfaces are curved and truncate other facies or cosets.	Migration of three-dimensional dunes.
Sp	Planar cross-bedded sandstone	Fine- to coarse-grained sandstones in which the foresets dip between 15° and 35°.	Migration of two-dimensional dunes.
Sr	Ripple cross-laminated sandstones	Very fine- to medium-grained sandstones with ripple cross-laminations.	Lower flow regime, wave and wind induced ripples.
Sm	Massive or weakly graded sandstone	Very fine- to coarse-grained sandstones in which no internal sedimentary structures are observed.	Rapid deposition e.g., sediment gravity flows. Alternatively, loss of sedimentary structures couple be due to bioturbation and/ or weathering.

Sd*	Sandstone/siltstone beds with deformation structures	Fine-grained sandstone and siltstone beds that display soft-sediment deformation structures including load casts, ball and pillows, and water escape and flame structures.	Rapid sedimentation, liquefaction, reverse density gradation, shear stress, or collapse of channel banks.
R*	Rhythmically interbedded mudstones, siltstones, and sandstones	Alternating heterolithic beds of fine- to medium-, and occasionally coarse-grained sandstones, siltstones, and mudstones. Lower bounding surface of coarser beds is sharp, whereas the upper bounding surface commonly has symmetrical ripple marks.	Deposition in parts of the interdistributary bay proximal to subaqueous distributary channels. Finer-grained argillaceous beds were deposited through suspension settling during periods of lower energy while the interbedded coarser-grained beds were deposited under higher energy conditions.
Fl	Laminated fines	Relatively thick (> 0.5 m) successions of horizontally and ripple-cross laminated mudstones.	Suspension settling, or deposition under lower flow regime conditions. Depositional settings include distal interdistributary bays, lagoons, and on tidal flats; away from channels and where wave activity is minimal. Fluvial settings include ephemeral floodplain pools and ponds.
Fm	Massive siltstones and mudstones	Massive beds of siltstone and mudstone displaying no apparent bedding planes. Co-occurs with Facies Fl and thin sandstone lenses may be present.	Either through suspension settling, with bioturbation, diagenesis, or weathering resulting in the lack of sedimentary structures, or through rapid deposition e.g., hyperpycnal/hypopycnal flows.

Due to the large area of the palaeosurface and the relatively shallow impressions ~~which~~ are preserved, traditional casting methods were not successful. Consequently, we used high-resolution scanning to digitise and accurately record the palaeosurface. Combining white-light source photogrammetry and aerial images, we were able to accurately portrait the surface trackway.

An orthophotograph of the palaeosurface was created using 16 photographs taken with a DJI (Shenzhen, China) Spark unmanned aerial vehicle (UAV). The photographs were taken 9.8 m meters above the surface (f/2.6, 1/100s, at 25 mm focal length) using the integrated 1/2.3" CMOS (Effective pixels: 12 MP) sensor and gimble. Each photograph was automatically

GPS tagged using the UAV's built in GPS functionality and later stitched into the encompassing orthophotograph using Microsoft Image Composite Editor 2.0.

In addition to photographing the large traces, high-resolution three-dimensional models for six of the better-preserved large traces were produced by scanning using an Artec Eva (Luxembourg City, Luxembourg; http://www. artecthree-dimensional.com/hardware/artec-eva/) handheld white-light source scanner and processed in Artec Studio 10 and Artec Studio 11 software. The models were exported as Polygon File Format (.ply) and processed further using the software CloudCompare 2.9.1 (https://www.danielgm.net/cc/).

4 RESULTS

4.1 Sedimentology

The Dave Green ichnofossils are preserved on the upper bedding plane of a ~10 cm thick ripple-marked fine-grained sandstone bed with a thin (~2 mm) mudstone drape, which we interpret to be situated in the upper part of the Waterford Formation (Fig. 4).

Fig. 4. Figure 4. Sedimentary structures preserved on the palaeosurface. A and B) Well preserved asymmetrical ripples indicate a westerly current direction. C) Current-modified ripples, with tongues of sand on the lee side of the ripples, in the northwestern part of the surface. D) Rill marks on smoothed ripple marks in the northwestern part of the surface. E) Depressions with interference ripples F) Smooth, unrippled section in the foreground with rippled surface in the background and a 'corridor' of circular depressions (tracks) between the two. G) Aerial view of the portion of the 'corridor' passing between impressions 2 and 3. H) Closer view of smooth areas surrounded by ripple marked depressions which could indicate the presence of a microbial mat on the surface, as seen in modern environments (I and J). I) Modern example of smooth areas surrounded by ripple marked depressions at the Mbotyi River Mouth, South Africa. J) Modern example of an algal mat and ripple patches from Mellum Island tidal flats, Germany (modified from Gerdes 2007).

The exposed palaeosurface is formed in a succession of interbedded mudstones, siltstones, and sandstones in the upper 0.5 m of the Waterford Formation, that are in turn overlain by mudstones and sandstones of the Balfour Formation (Fig. 1). Preserved asymmetrical ripples are well defined (Fig. 4A and B) with sinuous crestlines and well-developed bifurcations, especially in the western half of the surface. The ripples have a wavelength of 35 mm and an amplitude of 3 mm. Palaeocurrent readings from the asymmetrical ripples indicate a westerly

current direction of 258°. Current-modified and rounded, smooth-topped ripples with rill marks and *Gyrochorte*-like invertebrate traces are present on the northwestern part of the surface (Fig. 4C and D).

Several medium-sized depressions (>20 cm diameter) contain symmetrical ripples with a strike orientation of 278°, almost perpendicular to that of the asymmetrical ripples (Fig. 4E). Large, smooth areas surrounding asymmetrically ripple-marked sections (Fig. 4F and H) were observed in many parts of the surface; in such areas, no ripples are preserved, or the amplitude of the ripples is much smaller, while the wavelength remains the same. Numerous smaller, circular depressions, many with asymmetrical ripples, are also preserved (Fig. 4F and G) and are described further below under Circular depressions.

4.2 Ichnology

The exposed palaeosurface is covered with numerous traces and tracks of various size and form (Fig. 5). For the purposes of this paper, we will describe and discuss the different tracks in three groups: “Large impressions”, “Closely grouped depressions”, and “Linear traces”.

Fig. 5. Sketch map of the palaeosurface showing the location of the seven large impressions, smooth areas and linear traces. The palaeocurrent as indicated by the asymmetrical ripples is 258°. The six large impressions are numbered 1 – 7 and this scheme is followed in the text and subsequent figures. The linear traces are labeled LT1 – LT3

4.2.1 Large impressions

At least seven large elongate and spindle-shaped impressions are preserved on the surface (Figs. 2, 5 and 6; Table 2). These impressions vary in length between approximately 1.5-2.1 m, and the shape of each trace may be described in three sections, namely: 1) a tapered “tail” opening towards, 2) an expanded intermediate region (present in impressions 3, 4, 5), and 3) a wide rounded rectangular “body” between 200 mm and 220 mm wide (Fig. 6). The outer edge of the impressions manifest as pushed-up ridges or bulges. Some of the impressions are followed or preceded by smoothed traces 120 – 190 mm wide and up to several metres long, which generally interrupt or obliterate the ripples. These smooth traces connect some of the

impressions (e.g., impressions 3, 4, 5) and reveal two large semi-circular patterns (Fig. 5). Vague sinusoidal patterns are present in some of the smooth traces e.g., impressions 3 and 5.

Fig. 6. Oblique field photographs of the six best preserved large impressions at the Dave Green ichnosite and simplified sketch of an impression showing the characteristic “body”, “tail”, and intermediate expanded sections. Numbering of the impressions corresponds with the rest of the figures and text. In parts 1–5: scale bars equal 30 cm.

Impression 2 is 3.15 m long and consists of two superimposed impressions, with the tail section of the second impression (B) overprinting on the body section of the first (A; Fig. 7). The length of Impression 2A is ~2.09 m, whereas that of impression 2B is ~1.52 m. The width for the body portion of the impressions varies between ~180 and 200 mm, with the expanded section in impression 2B attaining a width of ~230 mm. A low ridge is present along the midline of impression 2 (Fig. 7).

Fig. 7 Details of impression 2. Textured scan (Left) with the outline of the impressions [2A (yellow) and 2B (white)] and footprints compared to the false-colour depth model (Right). Depth scale is in mm.

Table 2. Dimensions of the better-preserved large impressions and selected smooth traces.

**Trace**	**Length (m)**	**Width (mm)**
Impression 2A	2.09	180-200
Impression 2B	1.52	180-230
Impression 3	1.59	180-210
Impression 4	1.61	150-180
Impression 5	1.59	180-210
Smooth trace following impression 3	-	120-130
Smooth trace following impression 4	-	195-200
Smooth trace following impression 5	-	135-185
Smooth trace following impression 7	-	120-145

At least 15 round depressions, interpreted as footprints, are preserved between 90 and 160 mm away from the body impressions in impression 2 (Fig. 7). Eleven of these are alongside impression 2A and four of them alongside impression 2B. The lack of morphology in the

footprints makes it difficult to distinguish manus from pes. However, there are only four footprints adjacent to impression 2B, with the posterior pair of footprints positioned between 530 and 590 mm from the anterior pair for the right and left, respectively. A similar distance separates the posterior from the anterior pair of footprints in impression 2A. This most probably corresponds to the gleno-acetabular distance, which is reported to be a good estimate of the body length of a trace-maker (Padian & Olsen 1984; Schneck & Fritz 1985; Thulborn 1990). As such, we estimate the body length of the trace-maker to be approximately 560 mm.

Impression 3 (Fig. 6 and S1) has a length of ~1.59 m and a body width of between 180 and 210 mm, pinching down to ~140 mm at the juncture between the body and expanded intermediate section. A low ridge is present in the centre of the expanded intermediate section and near the point where the tail and intermediate portions join. Impression 3 leads into a 120–130 mm wide smooth trace (Fig. S1) and is cut by a second smooth trace that is 120–145 mm wide and which crosses in front of impression 3. A feint impression (impression 7) is present near the inferred start of this second smooth trace (Fig. S1).

Impression 4 (Fig. 6 and S2) is 1.61 m long, and the width of the body portion varies between 150 and 180 mm. It is also followed by a ~195–200 mm wide smoothed trace.

Impression 5 (Fig. 6 and S3) is ~1.59 m long and 180–210 mm wide in the body section, narrowing to about 160 mm at the point between the body and intermediate portions. A small, ovoid depression (~38 mm wide, ~60 mm long), possibly a footprint, is present 125 mm to the right and near the back of the body impression (Fig. S3). A low ridge is present along the midline of the impression and is most prominent around the juncture between the tail and intermediate portions. Impression 5 is followed by a wavy swim trace 135–185 mm wide (Fig. 6 and S3).

4.2.2 Circular depressions

Several smaller, circular depressions (Fig. 4F and G) occur in associated groupings. Three such groupings, indicated in Fig. 5 as “Smooth area with depressions” since the area surrounding the depressions is often smooth and not rippled, are: 1) a higher density of these depressions preserved in the northwestern part of the surface; 2) a concentration just north of the present-day island; and 3) a “corridor” ~0.9 m wide that crosses the surface from the western to eastern side (Fig. 4G). The depressions vary in size with diameters ranging from 10–15 cm. The bottom of most of the depressions is sculpted with asymmetrical ripples and little-to-no morphological details are preserved.

4.2.3 Linear traces

Several examples of single or paired linear traces are preserved on the palaeosurface. These traces are not very deep and are most readily observed during the low-angle light of early morning. Three of these, LT1, LT2, and LT3, are indicated on the map (Fig. 5).

The first linear trace (LT1) consists of a paired trace which begins along the eastern side of the palaeosurface (Fig. 8A) and heads westward across the surface before making a sharp turn (Fig. 8C and D) and heading northwestwards. The individual traces are ~1 cm wide and the paired traces are ~35 cm apart. Except for a short section, the trails are relatively straight and lack the typical sinuous shape normally associated with fish trails. In the sinuous section (Fig. 8B), the trails have a wavelength of 36–39 cm and an amplitude of 5 cm. The trace can be followed to the northwestern edge of the surface where it overprints Impression 1 (Fig. 8E).

Fig. 8. Linear traces preserved on the palaeosurface. A-E) A large ~~pairedlarge~~, paired trace (LT1) can be followed from the eastern edge of the surface (A) and is mostly straight except for a small sinuous section (B). The trace makes a sharp turn (C and D) and heads northwestwards, overprinting Impression 1 (E). The box outlined in A is the area shown in B. Hammer for scale. F) Paired trace (LT2) cutting through ripple crests only. The trace crosses the smoothed swim trail in the Southwestern corner of the surface. G) Long linear trace (LT3) on northern part of the surface. H) *Undichna* trace in north-western part of the surface (Off mapped area). Black arrows show unidentified invertebrate traces. Numbers on scale card are in cm. I) Linear paired trace cutting ripple crests in north-western part of the surface (Off mapped area).

A second linear trace (LT2) is present in the southeastern corner of the surface. LT2 consists of an approximately 7 cm wide paired straight trace that can be followed for roughly 4 m and crosses the smooth trace connecting Impressions 5 and 6 (Fig. 8F). LT2 affects the tops of the ripple crests but not the troughs.

A third linear trace (LT3) cuts across the northern portion of the surface. Although faint, this long unpaired trace is almost 8 m long and disrupts both the ripple crests and troughs (Fig. 8G).

Northwest of the mapped palaeosurface, a further two linear traces were observed on the rippled sandstone surface. The first of these is a ~3 mm wide sinuous trace with a wavelength of ~3 cm and amplitude of ~2 cm (Fig. 8H). The size of the trace allows for it to be attributed to *Undicna unisulca* and not *Cochlichnus*, as differentiated by Minter and Brady (2006). The second is a straight paired trace, with the two ~3 mm wide traces 3 cm apart (Fig. 8I). This trace disturbs the ripple crests and some of the troughs.

5 DISCUSSION

5.1 Depositional setting

Based on sedimentological field observations, we interpret the Dave Green palaeosurface to represent a sandy tidal flat or the sandy floor of a shallow embayment or lagoon. The traces made by fish, which overprint ~~which~~ some of the large impressions and are therefore younger, indicate that the palaeosurface was submerged at the time that the traces were made and *Gyrochorte*-like traces in the northwestern part of the surface provide evidence for a moderate energy, near-shore to shallow marine environment (de Gibert & Benner 2002). The presence of unrounded asymmetric ripple crests, with the exception of some ripples in the northwestern part of the exposure, suggest that the surface was not reworked and rill marks on the surface of some of the smoothed ripples (Fig. 4D) indicate the subsequent fall in water

level leading to subaerial exposure of at least the ripple crests (Reineck & Singh 1975; Selley 1985; Stow 2011). However, that the surface did not dry out completely before being buried by mud is suggested by the absence of desiccation cracks or other evidence for extended exposure.

Depressions on the mapped surface that contain symmetrical ripples (Fig. 4E) with crest strike orientations similar to the oscillatory ripples from other parts of the section (Groenewald 2015, 2021), but differing from that of the asymmetrical ripples by around 70°, could have formed as erosional depressions, or possibly footprints, which remained filled with water following the drop in water level. Wind blowing across the surface of these water-filled depressions could have resulted in the formation of the symmetrical ripple marks.

The smoother, non-rippled areas of the surface are possibly where a biofilm or microbial mat was growing on the sand in a shallow water environment similar to such mats that can be seen on present day tidal flats (Fig. 4I and J) (Cuadrado *et al.* 2012; Gerdes 2007; Reineck & Singh 1975; Schieber 1998; Selley 1985). The transition between the rippled and non-ripples areas is smooth, indicating that the surfaces are penecontemporaneous (Schieber 1998). Tracks and traces require the substrate to be 'just right' in order to be preserved (Falki[illegible]am *et al.* 2014) and microbial mats are commonly associated with fossil trackways, including in the Karoo (Marsicano *et al.* 2014; Sciscio *et al.* 2016, 2020; Smith 1993). In the case of the Dave Green Palaeosurface, microbial mats likely stabilised the surface sediments, preventing the formation of ripple marks in the smooth areas, and could have also influenced the mineralization of the drape on the surface (Cuadrado *et al.* 2012; Gerdes 2007).

5.2 Potential tracemakers

5.2.1 Large impressions

The seven large impressions preserved on the palaeosurface are interpreted to be body and tail impressions formed as temporary resting traces (cubichnia; Seilacher 2007), whereas the smoothed linear traces are interpreted as locomotory (swim) traces (repichnia; Seilacher 2007). Superficially, the large impressions preserved on the palaeosurface are similar in appearance to some present-day examples produced by crocodiles and alligators (Fig. 9A)(Farlow *et al.* 2018a; Milàn & Hedegaard 2010). However, crocodylomorphs do not appear in the fossil record until the Upper Triassic/ Early Jurassic (Clark *et al.* 2004; Colbert 1952; Colbert & Mook 1951; Crush 1984; Dollman & Choiniere 2022; Lockley *et al.* 2010; Parrish 1991) and the earliest crown-group crocodilians are from the Late Cretaceous, or possibly a little older (Brochu 2003; Lockley *et al.* 2010; Schwab *et al.* 2020). Since the Dave Green ichnofossils are late Permian, it is highly improbable that the traces were made by a crocodylomorph. The only plausible candidate for the maker of the large impressions and traces is a rhinesuchid temnospondyl, since this clade is the only group of tetrapods with a “crocodile-like” body plan known from the *Daptocephalus* AZ, and indeed the immediate vicinity of the study area (Eltink *et al.* 2019; Marsicano *et al.* 2017; Tarailo 2018; Viglietti 2020).

Fig. 9. A) Large saltwater crocodile with associated tail drag on a tidal flat in Australia shown next to impression 2 from the palaeosurface. (Crocodile Photo: animals.nationalgeographic.com/animals/ reptiles/saltwater-crocodile). B) A rhinesuchid temnospondyl such as *Laccosaurus* or *Uranocentrodon* is the probably the trace-maker.

Based on the preserved impressions, the tail and body of the trackmaker was ~ 1.59 m long. Adding a skull with a length of ~30 cm, typical for late Permian rhinesuchids, gives a total length of 1.89 m for the tracemaker. This is within the size of *Uranocentrodon senekalensis* (Fig. 9B), which has a skull length of up to 50 cm and a total adult body length of between 2.3 and 3.75 m (van Hoepen 1915). *Laccosaurus watsoni* is slightly smaller with a skull

length of approximately 23 cm for the holotype (Haughton 1925) but too few specimens are reliably attributed to this genus to estimate it's maximum body size.

Body impressions of amphibians showing most or all of the body outline are rare, with only three named ichnospecies described from the Palaeozoic, *Hermundurichnus* (Walter and Werneburg 1988) and *Sauropleura* (Langiux 1980; Langiux *et al.* 1974) from the Carboniferous-Permian of Europe (Langiux 1980; Langiux *et al.* 1974; Walter and Werneburg 1988 in Lucas *et al.* 2010 and Fillmore *et al.* 2012;) and *Temnocorpichnus isaacleai* from the late Mississippian of eastern Pennsylvania (Acton 2012; Fillmore *et al.* 2012, 2013; Lucas *et al.* 2010). Amphibian body resting traces associated with trackways and swimming traces have previously been described by Turek (1989), but were not named. All these examples are, however, as much as 10 times smaller than the impressions reported here.

While some species of stereospondyls, including rhinesuchids such as *Uranocentrodon senekalensis*, had ventral osteoderms that were likely covered by a horny keratin layer (Findlay 1968; Schoch & Milner 2000), the studied impressions generally have smooth bottoms and do not show any definitive evidence for the trace maker having ventral osteoderms. Consistency or grain size of the substrate (Milàn 2003), or movement of the animal, smearing the trace and thereby destroying the impressions, could account for the lack of fine morphological detail in the traces. Alternatively, growth of a microbial mat on the substrate could have resulted in the loss of morphological detail over time (C[illegible]rado *et al.* 2012; Gerdes 2007; Reineck & Singh 1975, 1980; Schieber 1998; Selley 1985).

5.2.2 Circular depressions

We interpret the numerous smaller, circular depressions on the surface as likely representing footprints made by a group or several groups of medium-large terrestrial tetrapods that waded and crossed through the water, rather than being erosional depressions. This is based on the

grouping of the depressions as well as the ‘corridor’ that crosses the surface. The lack of morphology in the depressions is an indication of the fluid, liquified character of the substrate (Milàn 2003, 2006). The thin upper layer of the palaeosurface comprises a finer grained, mineralised drape that was capable of preserving smaller traces such as fish fin traces and *Cochlichnus* traces, but the walls of deeper depressions penetrating the more liquefied underlying substrate collapsed. The act of larger animals walking on the substrate would have torn the microbial film, exposing the sand underneath while the current was strong enough to form ripples in the base of these depressions. This indicates that these trackways were probably made before the temnospondyl traces. The most likely candidate for the makers of these trackways are dicynodont therapsids.

5.2.3 Linear traces

The ichnotaxon *Undichna* is used to describe traces that are sinusoidal or scalloped, single, paired, or multiple sets of overlapping grooves that form regular, repeating patterns in bottom sediments of aquatic environments (Anderson 1976; Minter & Braddy 2006; Seilacher 2007; Trewin 2000). These patterns are thought to have been made by pectoral, pelvic, anal, and caudal fins of fish dragging along the substrate during swimming behaviour (Anderson 1976; Hasiotis *et al.* 2007; Seilacher 2007; Trewin 2000). Differences in the amplitude and wavelength of these patterns are related to the fish size and shape, direction and velocity of the water current, and swimming speed (Trewin 2000). As such, we interpret the linear and sinusoidal traces seen on the palaeosurface as likely being created by fish of various shapes, sizes, and swimming styles. While most the traces are straight and lack the sinusoidal pattern characteristic of *Undichna*, this could be explained by the fish drifting with the current, or swimming with thunniform or ostraciform motion, resulting in the much lower amplitude of the pectoral fins and, thereby, of the trace itself. The sharp turn seen in LT1 could have been avoidance behaviour of the fish. It is also possible, however, that some of the more linear

examples were created by a stick drifting and dragging along the sediment, thereby forming a pseudo trace fossil (R. Gess, pers. comm. 2015).

5.3 Rhinesuchid locomotion

The trackway preserved on the Dave Green palaeosurface provides evidence for both swimming and bottom walking behaviours. Temnospondyl locomotion has traditionally been compared with extant examples of salamanders, newts, and crocodilians, since these are considered to have similar bauplans – sprawling gait with limbs of equal size, elongated trunk, and a tail – and are capable of both aquatic and terrestrial locomotion (Ashley-Ross *et al.* 2009; Ashley-Ross & Bechtel 2004; Marsicano *et al.* 2014; Mujal & Schoch 2020). Work by Marchetti (2019) and Marsicano et al. (2014) has suggested that large temnospondyls used mainly their forelimbs rather than hindlimbs for propulsion during terrestrial locomotion, as a consequence of their relatively larger skull, heavier pectoral girdle, and relatively reduced pelvic girdle and tail.

When considering the locomotory capabilities of stereospondyls, a number of different categories, ranging from terrestrial crawlers to fully aquatic swimmers, are recognized (Schoch & Milner 2000). With the exception of some terrestrially adapted species e.g. *Lydekkerina huxleyi* (Canoville & Chinsamy 2015; Pawley & Warren 2004), most stereospondyls have small limbs with rudimentarily ossified humeri, femora and girdle elements. This suggests limited terrestrial locomotory ability and most stereospondyls are therefore thought to have been aquatic (Pawley & Warren 2004; Schoch & Milner 2000), an interpretation further supported by histological studies (McHugh 2014). Some forms were adept swimmers, with a long, laterally compressed tail probably providing propulsion and the gracile forelimbs being used more for manoeuvring than active swimming (Schoch & Milner 2000) or when performing bottom-walking behaviour in a manner similar to extant crocodiles (Farlow *et al.* 2018b; Mujal & Schoch 2020). Others resembled the modern cryptobranchid

salamanders in body proportions and were likely less active swimmers but sufficiently agile to lunge and capture larger prey items (Schoch & Milner 2000; Wild 1980).

The fact that many of the impressions are isolated and have no connecting trackways or swim traces (e.g., the gap between Impressions 2 and 3) is interpreted as evidence for subaqueous activity (Hasiotis *et al.* 2007) i.e. representing where the animal left the ground and either floated or swam for a short distance within the water column before making contact with the substrate again. Impression 2 (Fig. 7), with associated footprints, would have probably been created when the animal touched down and performed bottom walking behaviour. The absence of morphological detail in the footprints is likely because the sediment was under water and very soft (Milàn 2006), and can also be associated with the way that amphibians and other animals walk underwater, since their legs are not bearing the full weight of the body (Ashley-Ross *et al.* 2009; Brand 1996; Hasiotis *et al.* 2007; Turek 1989). It should be noted that variable footfall patterns and stride lengths have been reported for underwater walking, depending on the conditions and behaviour, e.g. similar but more variable stride length have been recorded in California newts walking underwater compared to on land (Ashley-Ross *et al.* 2009), whereas much greater paces and strides have been recorded for bottom walking crocodiles, especially when punting or carried by waves (Farlow *et al.* 2018b). Changes in the footprint trackway associated with impressions 2A and 2B could therefore reflect a shift from bottom walking, with a similar but more variable stride length to terrestrial pedestrianism in 2A, after which the trackmaker pushed up and floated a short distance before settling on the bottom – hence only leaving four footprints associated with Impression 2B.

Impressions 2 and 3 are relatively close together and appear to follow on from one another (Figs. 2 and 5). As such, we consider these to have been formed by the same individual. The direction of movement can be determined by looking at the tail to body direction. For

example, impression 2 indicates an animal that was moving in a south-easterly direction, as evinced by the tail thickening in the body direction as well as the slightly bulged area behind some of the footprints, indicating the direction that the animal pushed against the substrate.

Impressions 3 and 5 provide insight into the swimming locomotion of the temnospondyl. Upon entering the water crocodylomorphs progress with lateral undulations of the tail and body (McAllister 1991; Seebacher *et al.* 2003), tucking their limbs next to the body to reduce drag (McAllister 1991). A similar swimming motion can be seen in extant salamanders (D'Août & Aerts 1997; Turek 1989). The expanded area present anterior to the tail in some of the impressions is interpreted as the area where the hind limbs of the trackmaker were tucked in next to the body while swimming. The swim trace preserves slight sinusoidal movements, which are interpreted as having been formed through continuous sub-undulatory propulsion (undulations that have no recovery stroke) in a manner similar to that observed in extant crocodiles (McAllister 1991; Seebacher *et al.* 2003) and salamanders (D'Août & Aerts 1997; Turek 1989).

Impression 3 shows one trace that is crossed and overprinted by a second trace (from impression 7) moving from west to east and which leads into a second large circular route that crosses the northern half of the palaeosurface before curving back towards impression 1 (Fig. 5). The large, semi-circular routes of the traces across the surface, and the associated orientation of the animal relative to the current along the route, with many impressions facing into or perpendicular to the inferred palaeocurrent direction, suggest that the animal was capable of swimming independently of the current. However, the absence of well-defined ripples in most of the body impressions in conjunction with the swim traces indicates that the current was no longer strong enough to overprint the traces with new ripple marks before the surface was buried and preserved by the overlying strata. Microbial mat growth could have also had an influence, especially with Impressions 2, 3 and 4 which occur closer to the

smooth areas where inferred microbial mat growth is most abundant, helping solidify the substrate and inhibiting the formation of ripple marks.

Some of the traces preserve sharp turns or changes in direction, e.g., impressions 3 and 4, suggesting that the tracemaker might have been an active hunter rather than being an ambush predator. It is plausible that the swim traces were made when the trace-maker was actively looking for and chasing its prey – possibly including fish. No observable footprint impressions or disturbances to the adjacent rippled surface are associated with the turns as observed in other amphibian swimming traces (Turek 1989), and the slightly enlarged area anterior to the tail implies that the hind limbs were tucked against the body. It is possible that the animal used its front legs to help steer in a manner similar to younger crocodiles (Seebacher *et al.* 2003) and that they did not touch the substrate. Another possibility is that the animal did not use its legs at all while swimming, as seen in larger crocodiles (Seebacher *et al.* 2003), but they were used only when the animal touched the substrate or while bottom walking (Farlow *et al.* 2018b; Mujal & Schoch 2020).

Although the presence of multiple body impressions on a single bedding plane in other localities has been interpreted as representing some type of gregarious, possible even mating behaviour– as suggested for *Temnocorpichnus isaacleai* (Lucas *et al.* 2010), we consider the impressions to represent the behaviour of one, maybe two individuals. This interpretation is based on the fact that many of the impressions appear to follow on from one another and are sometimes linked by the smoothed swim traces to form two circular routes. The second circular route overprints the first one at impression 3 and they were therefore made at different times. However, the lack of well-defined body impressions along the second route limits comparison of body measurements of the tracemakers and it is uncertain whether they were made by the same individual or by two separate individuals living in the same area.

6 CONCLUSIONS

The remarkable Dave Green palaeosurface preserves a number of unique trace fossils attributed to medium to large tetrapods, fish, and invertebrates that inhabited a predominantly sandy tidal flat or shallow sandy lagoon in the late Permian. The application and benefit of utilising digital recording methodologies, including 3D surface scanning and UAV photography of palaeosurfaces, is evident here. Among the many trace fossils are at least seven morphologically unique body impressions with associated swim traces, which we interpret as having been made by a medium to large rhinesuchid temnospondyl with a total length of approximately 1.89 m. These traces provide evidence for an active lifestyle of swimming and bottom-walking behaviour in rhinesuchid amphibians during the Changhsingian, possibly while looking for food or hunting. The preserved swim traces also support interpretations that rhinesuchid temnospondyls swam using an undulatory motion of the tail and held their legs tucked in next to the body in a manner similar to extant crocodiles.

7 ACKNOWLEGEMENTS

Thanks goes to Dr Natasha Barbolini and Dr Gideon Groenewald for assistance in the field and helpful discussions, and to Dave and Sandra Green for access to the site, assistance, and for providing accommodation for the field visits. Thanks also goes to Dr Billy de Klerk, Dr Rose Prevec and Dr Rob Gess for their valuable inputs. Opinions expressed and conclusions arrived at, are those of the authors and are not necessarily to be attributed to the NRF, the CoE-Pal, or to PAST.

8 AUTHOR CONTRIBUTIONS

DG: Formal Analysis, Funding Acquisition, Investigation, Visualisation, Writing – Original Draft Preparation, Writing – Review & Editing

AK: Investigation, Formal Analysis, Resources, Data Curation, Visualisation, Writing – Review & Editing

MD: Investigation, Supervision, Writing – Review & Editing

CP-C : Supervision, Writing – Review & Editing

JH: Investigation, Writing – Review & Editing

BR: Conceptualization, Funding Acquisition, Investigation, Resources, Supervision, Writing – Review & Editing

9 REFERENCES

ACTON, Q. A. 2012. *Issues in Global Environment: Biology and Geosciences.* 290 pp., Scholarly Editions, Atlanta, Georgia.

ANDERSON, A. M. 1976. Fish trails from the early Permian of South Africa. *Palaeontology* **19**(2), 397–409, pl. 54.

ANDERSON, A. M. 1981. The Umfolozia arthropod trackways in the Permian Dwyka and Ecca series of South Africa. *Journal of Paleontology* **55**(1), 84–108.

ASHLEY-ROSS, M. A. & BECHTEL, B. F. 2004. Kinematics of the transition between aquatic and terrestrial locomotion in the newt *Taricha torosa*. *Journal of Experimental Biology* **207**(3), 461–474. doi: 10.1242/jeb.00769

ASHLEY-ROSS, M. A., LUNDIN, R. & JOHNSON, K. L. 2009. Kinematics of level terrestrial and underwater walking in the California newt, *Taricha torosa*. *Journal of Experimental Zoology Part A: Ecological Genetics and Physiology* **311A**, 240–257. doi: 10.1002/jez.522

BORDY, E. M. 2021. Darting towards Storm Shelter: A minute dinosaur trackway from southern Africa. *South African Journal of Science* **117**(3/4), 94–104. doi: 10.17159/sajs.2021/9145

BORDY, E. M., LINKERMANN, S. & PREVEC, R. 2011. Palaeoecological aspects of some invertebrate trace fossils from the mid- to Upper Permian Middleton Formation (Adelaide Subgroup, Beaufort Group, Karoo Supergroup), Eastern Cape, South Africa. *Journal of African Earth Sciences* **61**, 238–244. doi: 10.1016/j.jafrearsci.2011.06.002

BORDY, E. M., RAMPERSADH, A., ABRAHAMS, M., LOCKLEY, M. G. & HEAD, H. V. 2020. Tracking the Pliensbachian–Toarcian Karoo firewalkers: Trackways of quadruped and biped dinosaurs and mammaliaforms. *PLOS ONE* **15**(1), e0226847. doi: 10.1371/journal.pone.0226847

BORDY, E. M., SZTANÓ, O., RAMPERSADH, A., ALMOND, J. & CHOINIERE, J. N. 2019. Vertebrate scratch traces from the Middle Triassic Burgersdorp Formation of the main Karoo Basin, South Africa: Sedimentological and ichnological assessment. *Journal of African Earth Sciences* **160**, 103594. doi: 10.1016/j.jafrearsci.2019.103594

BOTHA, B. J. V. & LINSTRÖM, W. 1977. Palaeogeological and palaeogeographical aspects of the upper part of the Karoo Sequence in North-Western Natal. *Annals of the Geological Survey of South Africa* **12**, 177–192.

BOTHA, B. J. V. & LINSTRÖM, W. 1978. A note on the stratigraphy of the Beaufort Group in north-western Natal. *Transactions of the Geological Society of South Africa* **81**, 35–40.

BRAND, L. 1996. Variations in Salamander Trackways Resulting from Substrate Differences. *Journal of Palaeontology* **70**(6), 1004–1010.

BROCHU, C. A. 2003. Phylogenetic approaches toward crocodylian history. *Annual Review of Earth and Planetary Science* **31**, 357–397.

CANOVILLE, A. & CHINSAMY, A. 2015. Bone Microstructure of the stereospondyl Lydekkerina huxley reveals adaptive strategies to the harsh post Permian-extinction environment. *The Anatomical Record* **298**(7), 1237–1254. doi: 10.1002/ar.23160

CISNEROS, J. C., DAY, M. O., GROENEWALD, J. & RUBIDGE, B. S. 2020. SMALL FOOTPRINTS EXPAND MIDDLE PERMIAN AMPHIBIAN DIVERSITY IN THE SOUTH AFRICAN KAROO. *PALAIOS* **35**(1), 1–11. doi: 10.2110/palo.2018.098

CLARK, J. M., XU, X., FORSTER, C. A. & WANG, Y. 2004. A Middle Jurassic 'sphenosuchian' from China and the origin of the crocodilian skull. *Nature* **430**, 1021–1024.

Colbert, E. H. 1952. A pseudosuchian reptile from Arizona. *Bulletin of the American Museum of Natural History* **99**, 561–592.

Colbert, E. H. & Mook, C. C. 1951. The ancestral crocodilian Protosuchus. *Bulletin of the American Museum of Natural History* **94**, 143–182.

Council for Geoscience 1998. 2828 Harrismith 1:250 000 Geological Map Series. Council for Geoscience, Pretoria, South Africa.

Crush, P. J. 1984. A late Upper Triassic sphenosuchid crocodilian from Wales. *Palaeontology* **27**, 131–157.

Cuadrado, D. G., Carmona, N. B. & Bournod, C. N. 2012. Mineral precipitation on modern siliciclastic tidal flats colonized by microbial mats. *Sedimentary Geology* **271–272**, 58–66. doi: 10.1016/j.sedgeo.2012.06.005

D'Août, K. & Aerts, P. 1997. Kinematics and efficiency of steady swimming in adult axolotls (Ambystoma mexicanum). *The Journal of experimental biology* **200**, 1863–1871.

Van Dijk, D. E. 1997. Insect Faunas of South Africa from the Upper Permian and the Permian/ Triassic Boundary. *Palaeontologia africana* **34**, 43–48.

Van Dijk, D. E. & Geertsema, H. 2004. A new genus of Permian Plecoptera (*Afroperla*) from KwaZulu-Natal, South Africa. *African Entomology* **12**(2), 268–270.

Dollman, K. N. & Choiniere, J. N. 2022. Palate evolution in early- branching crocodylomorphs: Implications for homology, systematics, and ecomorphology. *The Anatomical Record* **305**(10), 2766–2790. doi: 10.1002/ar.24993

Ellenberger, P. 1970. Les niveaux paléontologiques de premiere apparition des mammiféres primordiaux en Afrique du Sud et leur ichnologie: Establissement de zones stratigraphiques detaillées dans le Stormberg de Leshoto (Afrique du Sud)(Trias Superieur a Jurassique). *IUGS, 2nd symposium on gondwana stratigraphy and palaeontology* 343–370.

Ellenberger, P. 1972. Contribution à la classification des pistes de vertébrés du Trias: les types du Stormberg d'Afrique du Sud (I Partie). *Palaeovertebrata, Mémoire Extraordinaire* **1972**, 1–152.

Ellenberger, P. 1974. Contribution à la classification des pistes de vertébrés du Trias: les types du Stormberg d'Afrique du Sud (Hème partie: le Stormberg Superieur — I. Le biome de la zone B/1 ou niveau de Moyeni: ses biocénoses). *Palaeovertebrata, Mémoire Extraordinaire* **1974**, 1–143.

Eltink, E., Schoch, R. R. & Langer, M. C. 2019. Interrelationships, palaeobiogeography and early evolution of Stereospondylomorpha (Tetrapoda: Temnospondyli). *Journal of Iberian Geology* **45**(2), 251–267. doi: 10.1007/s41513-019-00105-z

Falkingham, P. L., Hage, J. & Bäker, M. 2014. Mitigating the Goldilocks effect: the effects of different substrate models on track formation potential. *Royal Society Open Science* **1**(3), 140225–140234. doi: 10.1098/rsos.140225

Farlow, J. O., Robinson, N. J., Kumagai, C. J., Paladino, F. V., Falkingham, P. L., Elsey, R. M. & Martin, A. J. 2018a. Trackways of the American Crocodile (*Crocodylus acutus*) in Northwestern Costa Rica: Implications for Crocodylian Ichnology. *Ichnos* **25**(1), 30–65. doi: 10.1080/10420940.2017.1350856

Farlow, J. O., Robinson, N. J., Turner, M. L., Black, J. & Gatesy, S. M. 2018b. Footfall pattern of a bottom-walking crocodile (*Crocodylus acutus*). *PALAIOS* **33**(9), 406–413. doi: 10.2110/palo.2018.037

Fillmore, D. L., Lucas, S. G. & Simpson, E. L. 2012. *Ichnology of the Mississippian Mauch Chunk Formation, eastern Pennsylvania*. Bulletin 54. 136 pp., New Mexico Museum of Natural History and Science, Albuquerque.

Fillmore, D. L., Lucas, S. G. & Simpson, E. L. 2013. Ichnological diversity of the continental Mississippian Mauch Chunk Formation, eastern Pennsylvania. **In**: Lucas,

S. G., DiMichele, W. A., Barrick, J. E., Schneider, J. W. & Spielmann, J. A. (eds.) Bulletin 60., *The Carboniferous-Permian Transition*. New Mexico Museum of Natural History and Science, Albuquerque.

Findlay, G. H. 1968. On the structure of the skin in *Uranocentrodon* (*Rhinesuchus*) *senekalensis*, Van Hoepen. *Palaeontologia africana* **11**, 15–22.

Gastaldo, R. A. & Rolerson, M. W. 2008. Katbergia Gen. nov., a new trace fossil from the Upper Permian and Lower Triassic rocks of the Karoo Basin: implications for palaeoenvironmental conditions at the P/TR Extinction event. *Palaeontology* **51**(1), 215–229. doi: 10.1111/j.1475-4983.2007.00743.x

Geertsema, H., Van Dijk, D. E. & Van den Heever, J. A. 2002. Palaeozoic insects of southern Africa: a review. *Palaeontologia africana* **38**, 19–25.

Geological Survey 1981. 2928 Drakensberg 1:250 000 Geological Map Series. *Compiled by*: Linström, W., 1979. Government Printer, Pretoria, South Africa.

Gerdes, G. 2007. Chapter two: Structures left behind by modern microbial mats in their host sediments. **In**: Schieber, J., Bose, P. K., Eriksson, P. G., Banerjee, S., Sarkar, S., Alterman, W. & Catuneanu, O. (eds.) *Atlas of microbial mat features preserved within the clastic rock record*. Elsevier,.

De Gibert, J. M. & Benner, J. S. 2002. The trace fossil Gyrochorte: ethology and paleoecology. *Revista Espanola de Paleontologia* **17**(1), 1–12.

Green, D. 1997. *Palaeoenvironments pf the Estcourt Formation (Beaufort Groups), KwaZulu-Natal*. Msc Thesis, University of Natal, Durban,.

Groenewald, D. P. 2015. *Tetrapod trackways and the Ecca-Beaufort contact in the Estcourt district*. Unpublished Unpublished Honours research project, University of the Witwatersrand, Johannesburg, South Africa.

Groenewald, D. P. 2021. *A litho- and biostratigraphic analysis of the lower Beaufort Group in the distal sector of the main Karoo Basin, South Africa – implications for the depositional history of the distal foredeep to back-bulge basin*. Unpublished Unpublished PhD Thesis, University of the Witwatersrand, Johannesburg, South Africa.

Groenewald, D. P., Day, M. O., Penn-Clarke, C. R. & Rubidge, B. S. 2022. Stepping out across the Karoo retro-foreland basin: Improved constraints on the Ecca-Beaufort shoreline along the northern margin. *Journal of African Earth Sciences* **185**, 104389. doi: 10.1016/j.jafrearsci.2021.104389

Groenewald, G. H. 1989. Stratigrafie en sedimentologie van die Groep Beaufort in die noordoos-Vrystaat. *Bulletin van die Geologiese Opname van Suid-Afrika* **96**, 62.

Groenewald, G. H. 1990. The use of palaeontology in the correlation of lithostratographic units in the Beaufort Group, Karoo Sequence of South Africa. *Palaeontologia africana* **27**, 21–30.

Hasiotis, S. T., Platt, B. F., Hembree, D. I. & Everhart, M. J. 2007. The Trace-Fossil Record of Vertebrates. **In**: Miller, W. (ed.) *Trace Fossils: Concepts, Problems and Prospects*. 196–218. Elsivier, Amsterdam. doi: 10.1016/B978-044452949-7/50138-8

Haughton, S. H. 1925. Investigations in South African Fossil Reptiles and Amphibia (Part 13) - Descriptive Catalogue of the Amphibia of the Karroo System. *Annals of The South African Museum* **22**, 227–261.

Hobday, D. K. & Tavener-Smith, R. 1975. Trace fossils in the Ecca of northern Natal and their palaeoenvironmental significance. *Palaeontologia africana* **18**, 47–52.

Van Hoepen, E. C. N. 1915. Stegocephalia of Senekal, O.F.S. *Annals of the Transvaal Museum* **5**(2), 125–149.

Johnson, M. R. 1976. *Stratigraphy and Sedimentology of the Cape and Karoo Sequences in the Eastern Cape Province*. Phd thesis, Rhodes University, Grahamstown,.

JOHNSON, M. R., VAN VUUREN, C. J., VISSER, J. N. J., COLE, D. I., WICKENS, H. DE V., CHRISTIE, A. D. M., ROBERTS, D. L. & BRANDI, G. 2006. Sedimentary rocks of the Karoo Supergroup. **In**: Johnson, M. R., Anhaeusser, C. R. & Thomas, R. J. (eds.) *The Geology of South Africa*. 461–499. Council for Geoscience, Pretoria.

DE KLERK, W. J. 2002. A dicynodont trackway from the Cistecephalus Assemblage Zone in the Karoo, east of Graaff-Reinet, South Africa. *Palaeontologia africana* **38**, 73–91.

LACEY, W. S., VAN DIJK, D. E. & GORDON-GRAY, K. D. 1975. Fossil plants from the Upper Permian in the Mooi River district of Natal, South Mrica. *Annals of the Natal Museum* **22**(2), 349–420.

LANGIUX, J. 1980. Premi`eres observations a Morteru (St´ephanien de Blanzy-Montceau) ichnites, faune, flore. *Revue P´eriodique de Vulgarisation des Sciences Naturelles et Historiques "La Physiophile" Montceau-le-Mines* **93**, 77–88.

LANGIUX, J., PARRIAT, H. & SCOTTY, D. 1974. Faune fossile du bassin de Blanzy- Montceau acquisitions r´ecentes. *Revue P´eriodique de Vulgarisation des Sciences Naturelles et Historiques "La Physiophile" Montceau-le-Mines* **80**, 55–67.

LINSTRÖM, W. 1987. Die Ecca/Beaufortkontak in sentraal- en suid-Natal. *Symposium on stratigraphic problems relating to the Ecca-Beaufort Contact* 23–24.

LOCKLEY, M. G., LUCAS, S. G., MILÀN, J., HARRIS, J. D., AVANZINI, M., FOSTER, J. R. & SPIELMANN, J. A. 2010. The fossil record of crocodylian tracks and traces: an overview. **In**: Milàn, J., Lucas, S. G., Lockley, M. G. & Spielmann, J. A. (eds.) Bulletin 5., *Crocodyle Tracks and Traces*. New Mexico Museum of Natural History and Science, Albuquerque.

LUCAS, S. G., FILLMORE, D. L. & SIMPSON, E. L. 2010. Amphibian Body Impressions from the Mississippian of Pennsylvania, USA. *Ichnos* **17**, 172–176. doi: 10.1080/10420940.2010.502488

MACRAE, C. 1990. Fossil vertebrate tracks near Murraysburg, Cape Province. *Palaeontologia africana* **27**, 83–88.

MARCHETTI, L. 2019. Can undertracks show higher morphologic quality than surface tracks? Remarks on large amphibian tracks from the Early Permian of France. *Journal of Iberian Geology* **45**(2), 353–363. doi: 10.1007/s41513-018-0080-4

MARCHETTI, L., KLEIN, H., BUCHWITZ, M., RONCHI, A., SMITH, R. M. H., DE KLERK, W. J., SCISCIO, L. & GROENEWALD, G. H. 2019. Permian-Triassic vertebrate footprints from South Africa: Ichnotaxonomy, producers and biostratigraphy through two major faunal crises. *Gondwana Research* **72**, 139–168. doi: 10.1016/j.gr.2019.03.009

MARCHETTI, L., KLEIN, H., BUCHWITZ, M., RONCHI, A., SMITH, R. M. H., DE KLERK, W. J., SCISCIO, L. & GROENEWALD, G. H. 2020. Reply to discussion of "Permian—Triassic vertebrate footprints from South Africa: Ichnotaxonomy, producers and biostratigraphy through two major faunal crises" by Marchetti, L., Klein, H., Buchwitz, M., Ronchi, A., Smith, R.M.H., DeKlerk, W.J., Sciscio, L., and Groenewald, G.H. (2019). *Gondwana Research* **78**, 379–383. doi: 10.1016/j.gr.2019.08.002

MARSICANO, C. A., LATIMER, E., RUBIDGE, B. & SMITH, R. M. H. 2017. The Rhinesuchidae and early history of the Stereospondyli (Amphibia: Temnospondyli) at the end of the Palaeozoic. *Zoological Journal of the Linnean Society* **181**, 357–384. doi: 10.1093/zoolinnean/zlw032

MARSICANO, C. A., WILSON, J. A. & SMITH, R. M. H. 2014. A Temnospondyl Trackway from the Early Mesozoic of Western Gondwana and Its Implications for Basal Tetrapod Locomotion. *PLoS ONE* **9**(8), e103255. doi: 10.1371/journal.pone.0103255

MASON, R., RUBIDGE, B. & HANCOX, J. 2015. Terrestrial vertebrate colonisation and the Ecca- Beaufort boundary in the south-eastern main Karoo Basin , South Africa :

implications for Permian basin evolution. *South African Journal of Geology* **118**(2), 145–156. doi: 10.2113/gssajg.118.2.145

Mason, T. R. & Christie, A. D. M. 1986. Palaeoenvironmental significance of ichnogenus *Diplocraterion* Torell from the Permian Vryheid Formation of the Karoo Supergroup, South Africa. *Palaeogeography, Palaeoclimatology, Palaeoecology* **52**, 249–265.

McAllister, J. A. 1991. Dakota Formation Tracks from Kansas: Implications for the recognition of tetrapod subaqueous traces. **In**: Gillette, D. D. & Lockley, M. G. (eds.) *Dinosaur Tracks and Traces*. 343–348. Cambridge University Press,.

McHugh, J. B. 2014. Paleohistology and histovariability of the Permian stereospondyl *Rhinesuchus*. *Journal of Vertebrate Paleontology* **34**(1), 59–68. doi: 10.1080/02724634.2013.787429

Miall, A. D. 1996. *The geology of fluvial deposits: Sedimentary facies, basin analysis, and petroleum geology*. Springer,.

Miall, A. D. 2016. *Stratigraphy: A Modern Synthesis*. 454 pp., Springer,. doi: 10.1007/978-3-319-24304-7

Milàn, J. 2003. *Experimental Ichnology - experiments with track and undertrack formation using emu tracks in sediments of different consistencies, with comparisons to fossil dinosaur tracks.* Unpublished Msc Thesis (Unpublished), University of Copenhagen, Copenhagen.

Milàn, J. 2006. Variations in the morphology of emu (Dromaius novaehollandiae) tracks reflecting differences in walking pattern and substrate consistency: Ichnotaxonomic implications. *Palaeontology* **49**(2), 405–420. doi: 10.1111/j.1475-4983.2006.00543.x

Milàn, J. & Hedegaard, R. 2010. Interspecific variation in track and trackways from extant crocydilians. **In**: Milàn, J., Lucas, S. G., Lockley, M. G. & Spielmann, J. A. (eds.) Bulletin 51., *Crocodyle tracks and traces*. 15–30. New Mexico Museum of Natural History and Science, Albuquerque.

Minter, N. J. & Braddy, S. J. 2006. The fish and amphibian swimming traces Undichna and Lunichnium with examples from the Lower Permian of New Mexico, USA. *Palaeontology* **49**(5), 1123–1142. doi: 10.1111/j.1475-4983.2006.00588.x

Mujal, E. & Schoch, R. R. 2020. Middle Triassic (Ladinian) amphibian tracks from the Lower Keuper succession of southern Germany: Implications for temnospondyl locomotion and track preservation. *Palaeogeography, Palaeoclimatology, Palaeoecology* **543**, 109625. doi: 10.1016/j.palaeo.2020.109625

Muntingh, D. J. 1989. *Die Geologie van die Gebied Frankfort: Explanation of sheet 2728, scale1: 250 000.* 23 pp., Department of Mineral and Energy Affairs, Geological Survey of South Africa, Pretoria, South Africa.

Muntingh, D. J. 1997. Sedimentologie en stratigraphie van die Ecca-Beaufort-oorgang in die noordoostelike gedeelte van die hoof Karookom. *Bulletin van die Geologiese Opname van Suid-Afrika* **121**, 46.

Olsen, P. E. & Galton, P. M. 1984. A review of the reptile and amphibian assemblages from the Stormberg of southern Africa, with special emphasis on the footprints and the age of the Stormberg

Padian, K. & Olsen, P. E. 1984. The fossil trackway Pteraichnus: not pterosaurian, but crocodilian. *Journal of Palaeontology* **58**(1), 178–184.

Parrish, M. J. 1991. A new specimen of an early crocodylomorph (cf. Sphenosuchus sp.) from the Upper Triassic Chinle Formation of Petrified Forest National Park. *Journal of Vertebrate Paleontology* **11**, 198–212.

Pawley, K. & Warren, A. 2004. Immaturity vs paedomorphism: a rhinesuchid stereospondyl postcranium from the Upper Permian of South Africa. *Palaeontologia africana* **40**, 1–10.

Porchetti, S. D. & Nicosia, U. 2007. Re-Examination of Some Large Early Mesozoic Tetrapod Footprints from the African Collection of Paul Ellenberger. *Ichnos* **14**(3–4), 219–245. doi: 10.1080/10420940601049990

Raath, M. A., Kitching, J. W., Shone, R. W. & Rossouw, G. J. 1990. Dinosaur tracks in Triassic Molteno sediments: the earliest evidence of dinosaurs in South Africa? *Palaeontologia africana* **27**, 89–95.

Raath, M. A. & Yates, A. M. 2005. Preliminary report of a large theropod dinosaur trackway in Clarens Formation sandstone (Early Jurassic) in the Paul Roux district, northeastern Free State, South Africa. *Palaeontologia africana* **41**, 101–104.

Reineck, H. E. & Singh, I. B. 1975. *Depositional Sedimentary Environments*. 439 pp., Springer-Verlag, Berlin, Germany.

Reineck, H. E. & Singh, I. B. 1980. *Depositional Sedimentary Environments*. 551 pp., Springer-Verlag, Berlin, Germany. doi: 10.1007/978-3-642-81498-3

Rossouw, C. 2010. *Management proposals for the declaration of palaeontological and geological sites and the low impact adaptation of one of the sites on the farm Van der Merwes Kraal 972, Estcourt District.*

Rubidge, B. S., Hancox, P. . J. & Catuneanu, O. 2000. Sequence Analysis of the Ecca Beaufort contact in the Southern Karoo. *South African Journal of Geology* **103**(1), 81–96.

Rubidge, B. S., Hancox, P. J. & Mason, R. 2012. Waterford Formation in the south-eastern Karoo: Implications for basin development. *South African Journal of Science* **108**, 3–7. doi: 10.4102/sajs.v108i3/4.829

SACS, S. A. C. for S. 1980. *Stratigraphy of South Africa - Handbook 8 Part 1: Lithostratigraphy of the Republic of South Africa, South West Africa/Namibia and the republics of Bophuthatswana, Transkei and Venda*. Geological Survey, Pretoria.

Savage, N. M. 1971. A varvite ichnocoenosis from the Dwyka Series of Natal. *Lethaia* **4**(2), 217–233. doi: 10.1111/j.1502-3931.1971.tb01290.x

Schieber, J. 1998. Possible indicators of microbial mat deposits in shales and sandstones: examples from the Mid-Proterozoic Belt Supergroup, Montana, USA. *Sedimentary Geology* **120**(1), 105–124.

Schneck, W. M. & Fritz, W. J. 1985. An Amphibian Trackway (Cincosaurus cobbi) from the Lower Pennsylvanian ('Pottsville') of Lookout Mountain, Georgia: A First Occurrence. *Journal of Paleontology* **59**(5), 1243–1250.

Schoch, R. R. & Milner, A. R. 2000. *Stereospondyli: Stem-Stereospondyli, Rhinesuchidae, Rhytidostea, Trematosauroidea, Capitosauroidea. Handbuch der Paläoherpetologie (Encyclopedia of paleoherpetology),* Part 3B. 203 pp., Verlag Dr. Frederich Pfiel, München.

Schwab, J. A., Young, M. T., Neenan, J. M., Walsh, S. A., Witmer, L. M., Herrera, Y., Allain, R., Brochu, C. A., Choiniere, J. N., Clark, J. M., Dollman, K. N., Etches, S., Fritsch, G., Gignac, P. M., Ruebenstahl, A., Sachs, S., Turner, A. H., Vignaud, P., Wilberg, E. W., Xu, X., Zanno, L. E. & Brusatte, S. L. 2020. Inner ear sensory system changes as extinct crocodylomorphs transitioned from land to water. *Proceedings of the National Academy of Sciences* **117**(19), 10422–10428. doi: 10.1073/pnas.2002146117

Sciscio, L., Bordy, E. M. & Head, H. V. 2020. A Late Triassic aquatic community: *Undichna*-like and related swimming traces from a freshwater pond in the lower Elliot Formation of South Africa. *Journal of African Earth Sciences* **172**, 104026. doi: 10.1016/j.jafrearsci.2020.104026

Sciscio, L., Bordy, E. M., Reid, M. & Abrahams, M. 2016. Sedimentology and ichnology of the Mafube dinosaur track site (Lower Jurassic, eastern Free State, South Africa): a

report on footprint preservation and palaeoenvironment. *PeerJ* **4**, e2285. doi: 10.7717/peerj.2285

Seebacher, F., Elsworth, P. G. & Franklin, C. E. 2003. Ontogenetic changes of swimming kinematics in a semi-aquatic reptile (Crocodylus porosus). *Australian Journal of Zoology* **51**, 15–24.

Seilacher, A. 2007. *Trace fossil analysis*. 226 pp., Springer, Berlin.

Selley, R. C. 1985. *Ancient Sedimentary Environments*. 317 pp., Chapman and Hall, London.

Selover, R. W. & Gastaldo, R. A. 2005. A reinterpretation of the Wagendrift Quarry, Estcourt, KwaZulu-Natal Province, and its implications for Karoo Basin Paleogeography. *South African Journal of Geology* **108**(3), 429–438. doi: 10.2113/108.3.429

Smith, R. M. H. 1993. Sedimentology and Ichnology of Floodplain Paleosurfaces in the Beaufort Group (Late Permian), Karoo Sequence, South Africa. *PALAIOS* **8**(4), 339–357. doi: 10.2307/3515265

Smith, R. M. H., Eriksson, P. G. & Botha, W. J. 1993. A review of the stratigraphy and sedimentary environments of the Karoo-aged basins of Southern Africa. *Journal of African Earth Sciences (and the Middle East)* **16**(132), 143–169. doi: 10.1016/0899-5362(93)90164-L

Stanistreet, I. G., Le Blanc Smith, G. & Cadle, A. B. 1980. Trace fossils as sedimentological and palaeoenvironmental indices in the Ecca Group (Lower Permian) of the Transvaal. *Transactions of the Geological Society of South Africa* **83**, 333–344.

Stow, D. A. 2011. *Sedimentary Rocks in the field - a colour guide.* 320 pp., Academic Press, Burlingham, MA.

Tarailo, D. A. 2018. Taxonomic and ecomorphological diversity of temnospondyl amphibians across the Permian-Triassic boundary in the Karoo Basin (South Africa). *Journal of Morphology* **279**(12), 1840–1848. doi: 10.1002/jmor.20906

Thulborn, T. 1990. *Dinosaur Tracks*. 258 pp., Chapman and Hall, London, U.K.

Trewin, N. H. 2000. The ichnogenus Undichna, with examples from the Permian of the Falkland Islands. *Palaeontology* **43**(6), 979–997.

Turek, V. 1989. Fish and amphibian trace fossils from westphalian sediments of bohemia. *Palaeontology* **32**(3), 623–643, pls. 73–75.

Van Dijk, D. E. 1978. Trackways in the Stormberg. *Palaeontologia africana* **21**, 113–120.

Van Dijk, D. E., Channing, A. & Van Den Heever, J. A. 2002. Permian trace fossils attributed to tetrapods (Tierberg formation, Karoo basin, South Africa). *Palaeontologia africana* **38**, 49–56.

Viglietti, P. A. 2020. Biostratigraphy of the *Daptocephalus* Assemblage Zone. *South African Journal of Geology* **123**(2), 191–206.

Walter, H. & Werneburg, R. 1988. Über Liegesupren (Cubichnia) aquatischer Tetrapoden (?Diplocauliden, Nectridea) aus den Rotteroder Schichten (Rotliegendes, Thüringer Wald/DDR). *Freiberger Forschungsheft* **419**, 96–106.

Wild, R. 1980. Die Saurierfunde von Kupferzell. *Die Schwäbische Heimat (Stuttgart)* **31**(2), 110–117.

Wilson, J. A., Marsicano, C. A. & Smith, R. M. H. 2009. Dynamic Locomotor Capabilities Revealed by Early Dinosaur Trackmakers from Southern Africa. *PLoS ONE* **4**(10), e7331. doi: 10.1371/journal.pone.0007331

10 FIGURE CAPTIONS

Figure 1. Geological setting of the Dave Green palaeosurface. A) Simplified geological map of the main Karoo Basin. Position of the study area is indicated. B) Aerial view of the palaeosurface. C) Stratigraphic log measured along the Rensburgspruit.

Figure 2. Orthophoto of the palaeosurface with the positions of the seven large impressions and paths followed for the high-resolution scans indicated. Numbers used for the impressions are the same throughout the manuscript.

Figure 3. Features of the upper Waterford and lower Balfour formations in the study area. A) Large calcareous nodules. B) Rounded/ Smoothed symmetrical ripples on the top of the thick sandstone unit. C) Dark grey, laminated mudstones. D) Iron oxide haloes in sandstone unit. E) Soft sediment deformation. Note how the laminations follow the deformation bed. F) Sequence of rhythmites, comprising alternating mudstone and sandstone units. G) Large symmetrical ripples with sharp, straight crests and *Skolithos* burrows. H) Yellow-green mudstones typical of this facies association. I) Sandstone lens showing lateral/ downstream accretion. J) Calcareous concretions within a sandstone. K) Slickenside surfaces and nodules in mudstones. L) Fossilised wood weathering out of the mudstones. M) Horsetail impressions. N) *Glossopteris* leaf impressions. O) Large, unidentified bone c.f. dicynodont.

Figure 4. Sedimentary structures preserved on the palaeosurface. A and B) Well preserved asymmetrical ripples indicate a westerly current direction. C) Current-modified ripples, with tongues of sand on the lee side of the ripples, in the northwestern part of the surface. D) Rill marks on smoothed ripple marks in the northwestern part of the surface. E) Depressions with interference ripples F) Smooth, unrippled section in the foreground with rippled surface in the background and a ‘corridor’ of circular depressions (tracks) between the two. G) Aerial view of the portion of the ‘corridor’ passing between impressions 2 and 3. H) Closer view of smooth areas surrounded by ripple marked depressions which could indicate the presence of a microbial mat on the surface, as seen in modern environments (I and J). I) Modern example of smooth areas surrounded by ripple marked depressions at the Mbotyi River Mouth, South Africa. J) Modern example of an algal mat and ripple patches from Mellum Island tidal flats, Germany (modified from Gerdes 2007).

Figure 5. Sketch map of the palaeosurface showing the location of the seven large impressions, smooth areas and linear traces. The palaeocurrent as indicated by the asymmetrical ripples is 258°. The six large impressions

are numbered 1 – 7 and this scheme is followed in the text and subsequent figures. The linear traces are labeled LT1 – LT3

Figure 6. Oblique field photographs of the six best preserved large impressions at the Dave Green ichnosite and simplified sketch of an impression showing the characteristic “body”, “tail”, and intermediate expanded sections. Numbering of the impressions corresponds with the rest of the figures and text. In parts 1–5: scale bars equal 30 cm.

Figure 10 Details of impression 2. Textured scan (Left) with the outline of the impressions [2A (yellow) and 2B (white)] and footprints compared to the false-colour depth model (Right). Depth scale is in mm.

Figure 8. Linear traces preserved on the palaeosurface. A-E) A large pairedlarge, paired trace (LT1) can be followed from the eastern edge of the surface (A) and is mostly straight except for a small sinuous section (B). The trace makes a sharp turn (C and D) and heads northwestwards, overprinting Impression 1 (E). The box outlined in A is the area shown in B. Hammer for scale. F) Paired trace (LT2) cutting through ripple crests only. The trace crosses the smoothed swim trail in the Southwestern corner of the surface. G) Long linear trace (LT3) on northern part of the surface. H) *Undichna* trace in north-western part of the surface (Off mapped area). Black arrows show unidentified invertebrate traces. Numbers on scale card are in cm. I) Linear paired trace cutting ripple crests in north-western part of the surface (Off mapped area).

Figure 9. A) Large saltwater crocodile with associated tail drag on a tidal flat in Australia shown next to impression 2 from the palaeosurface. (Crocodile Photo: animals.nationalgeographic.com/animals/ reptiles/saltwater-crocodile). B) A rhinesuchid temnospondyl such as *Laccosaurus* or *Uranocentrodon* is the probably the trace-maker.

Figure S1. Details of impressions 3 and 7. Textured scan (top), with the outlines of the impressions and associated swim trails of impression 3 (White) and impression 7 (Yellow), shown next to the false-colour depth model (bottom). Depth scale is in mm.

Figure S2. Details of impression 4. Textured scan (Left) with the outline of the impression and swim trace shown next to the false-colour depth model (Right). Depth scale is in mm.

Figure S3. Details of impression 5. Textured scan (Left) with the outline of the impression and swim trace shown next to the false-colour depth model (Right). Depth scale is in mm.

11 TABLE CAPTIONS

Table 1. Descriptions and interpretations of lithofacies types [modified after Miall (2006); * denotes newly erected lithofacies types from Groenewald *et al.* (2022)] from the studied stratigraphic interval.

Table 3. Dimensions of the better-preserved large impressions and selected smooth traces.

Fig 1 Click here to access/download;Figure;Fig1.tiff

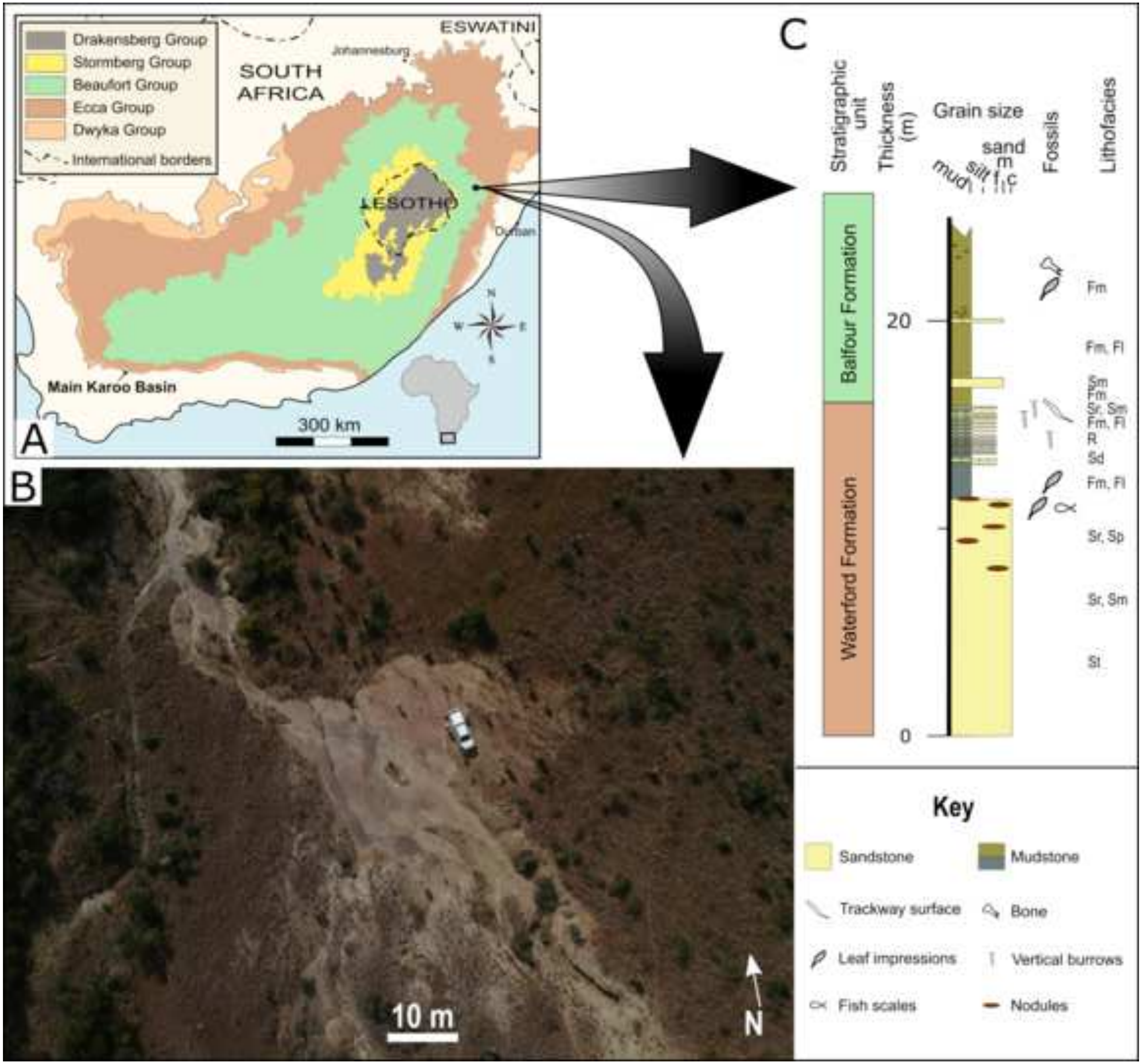

Fig1.tiff

Fig 2

Click here to access/download;Figure;Fig2.tiff

Fig2.tiff

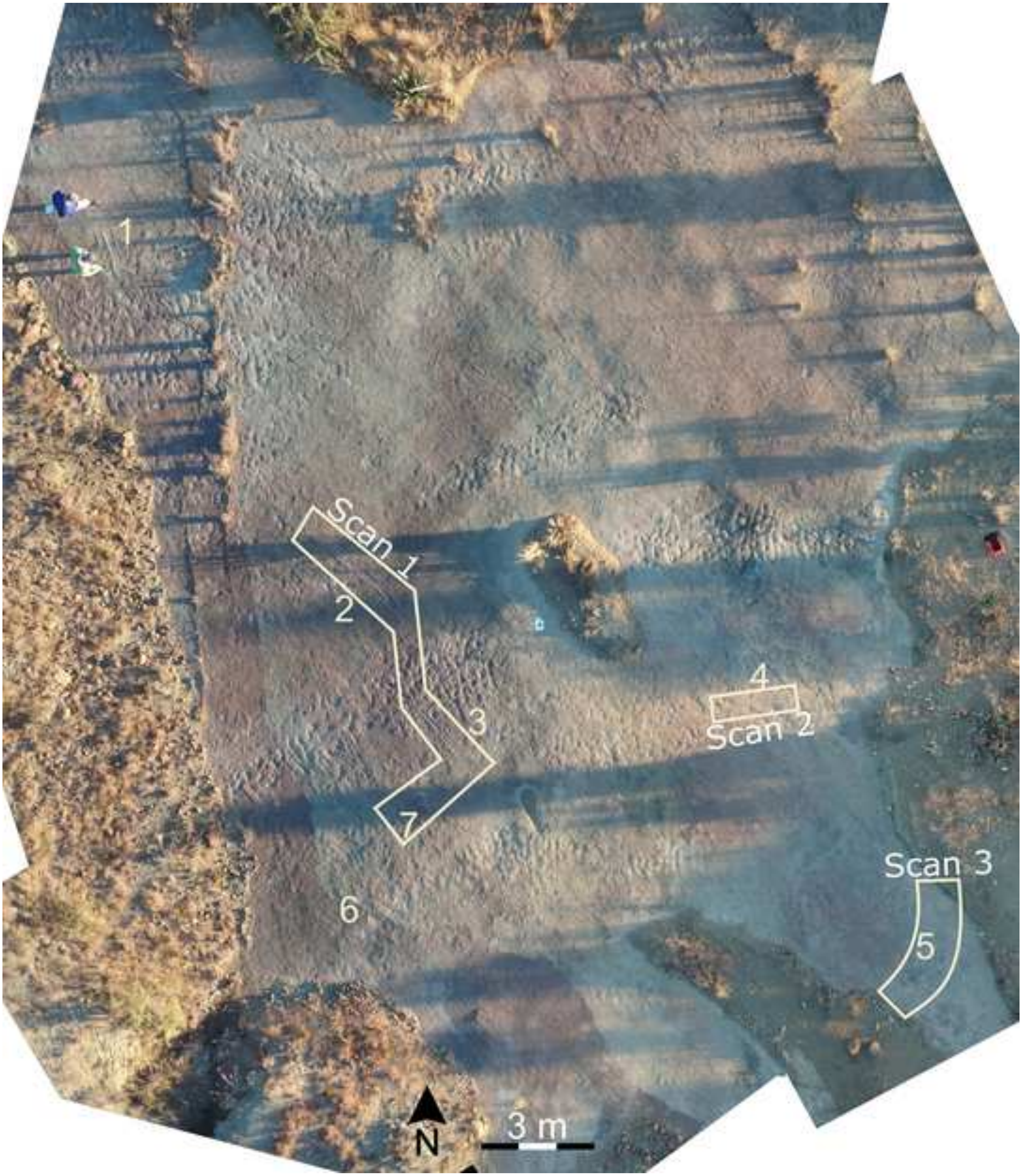

Fig 3

Click here to access/download;Figure;Fig3.tiff

Fig3.tiff

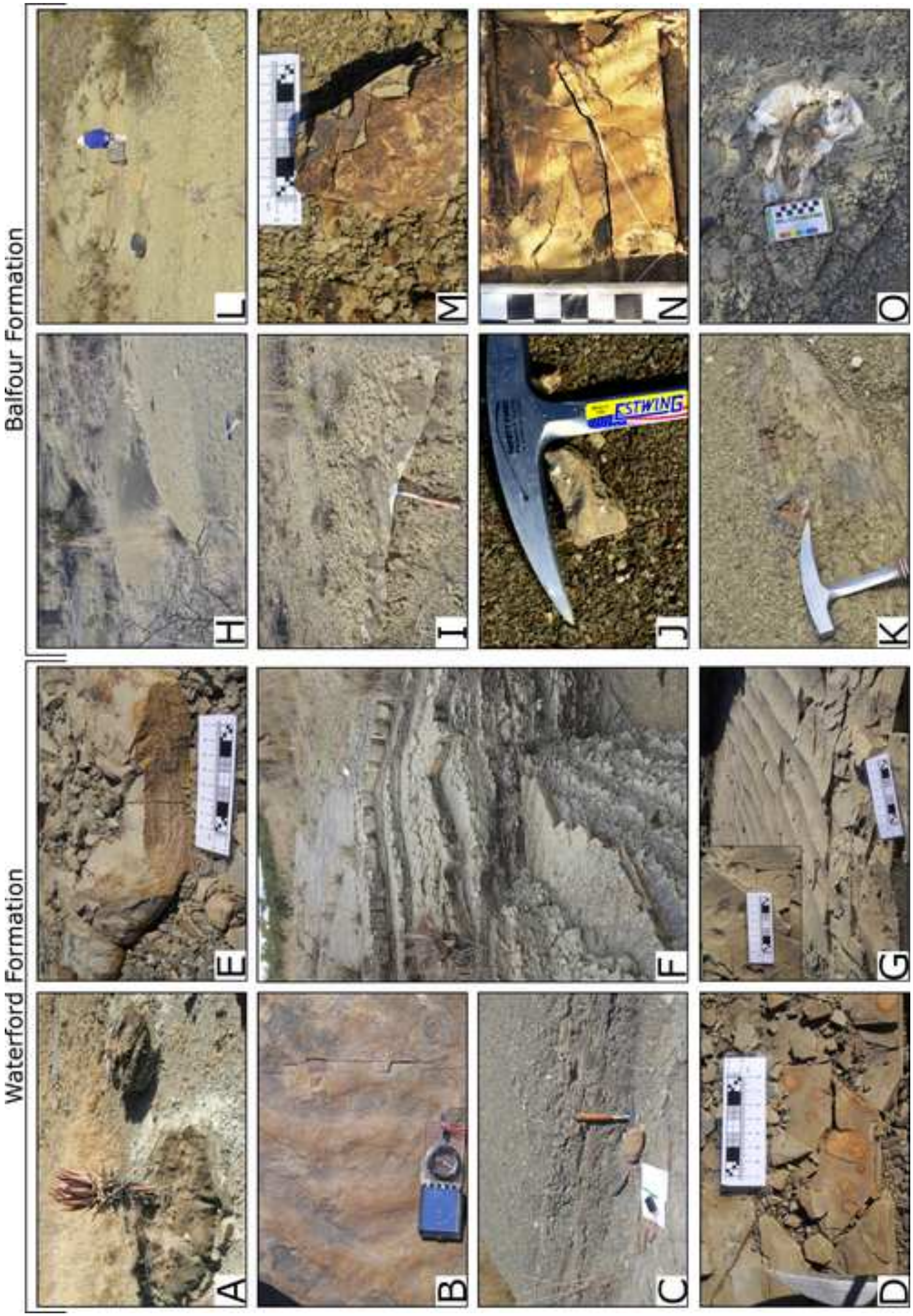

Fig 4 Click here to access/download;Figure;Fig4.tiff

Fig4.tiff

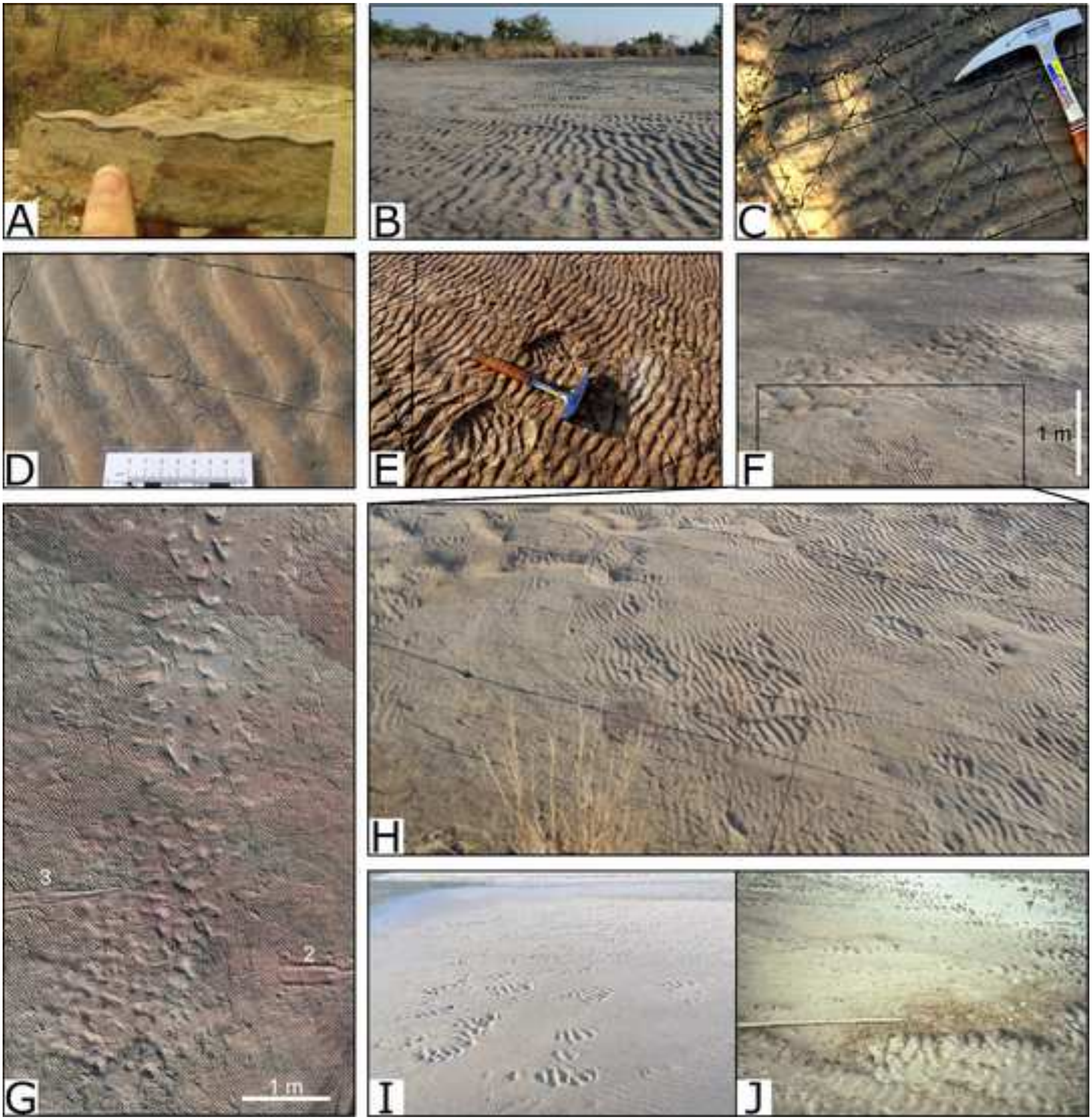

Fig 5
Click here to access/download;Figure;Fig5.tiff

Fig5.tiff

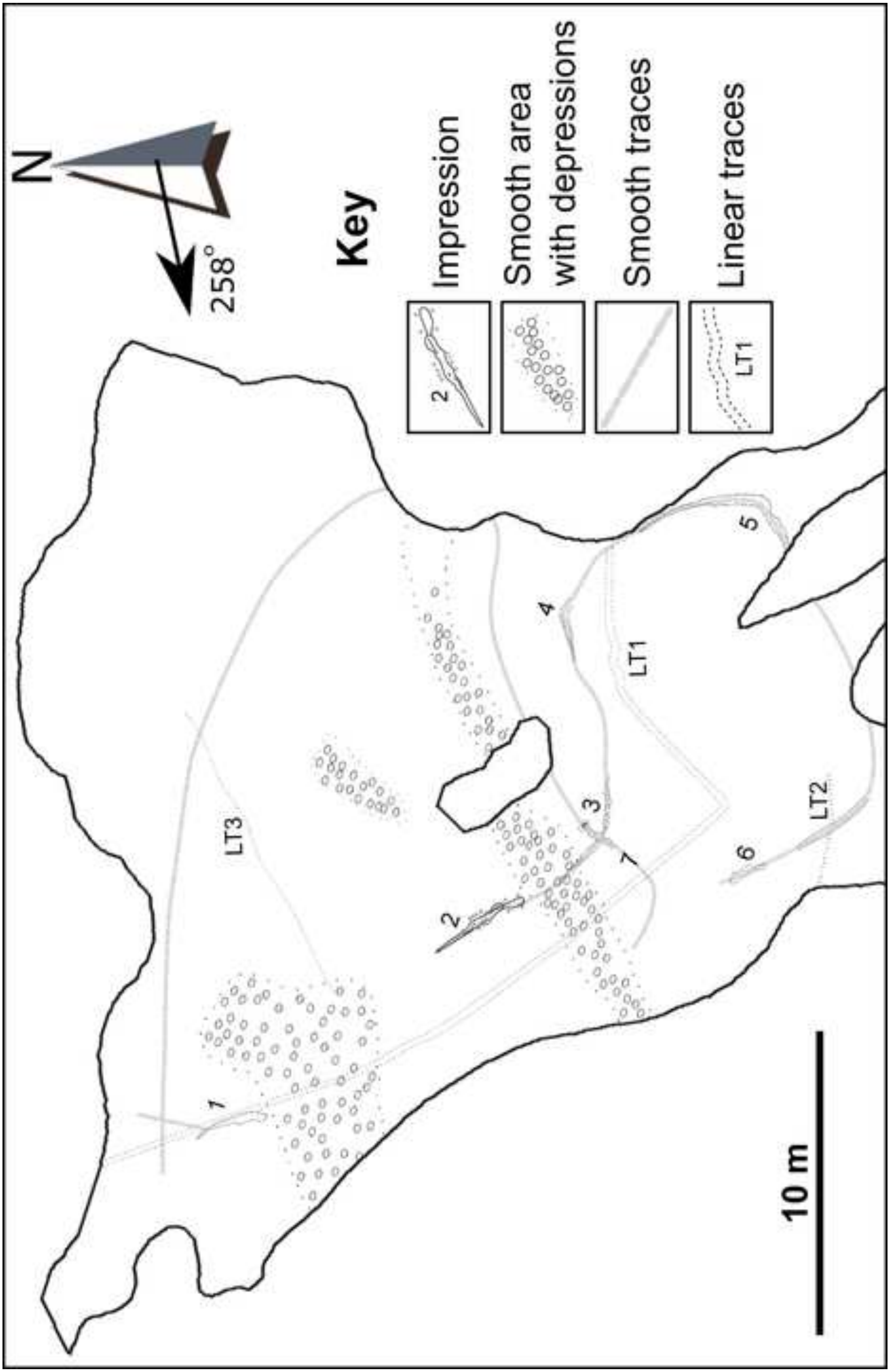

Fig 6

Click here to access/download;Figure;Fig6.tiff

Fig6.tiff

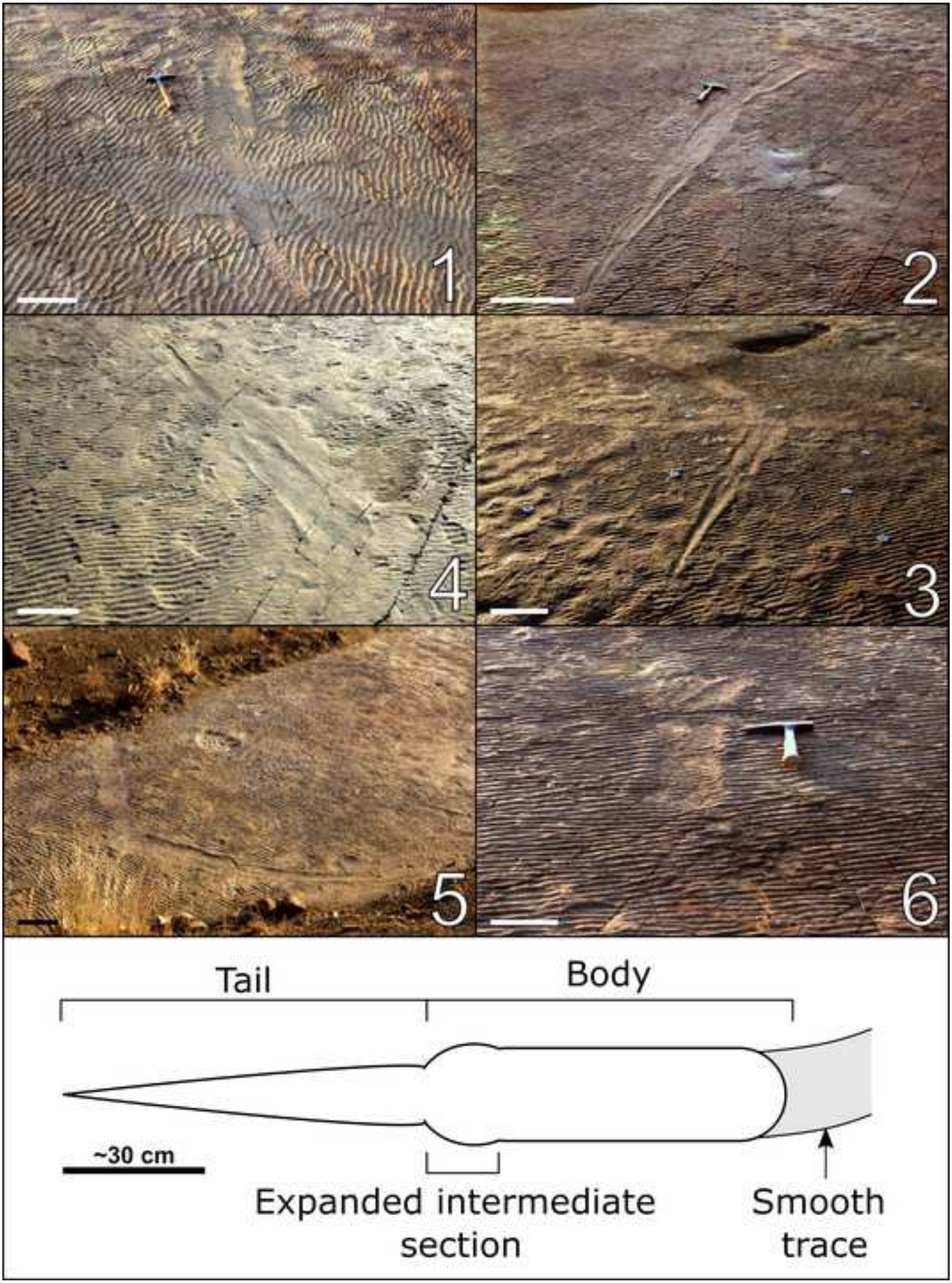

Fig 7

Click here to access/download;Figure;Fig7.tiff

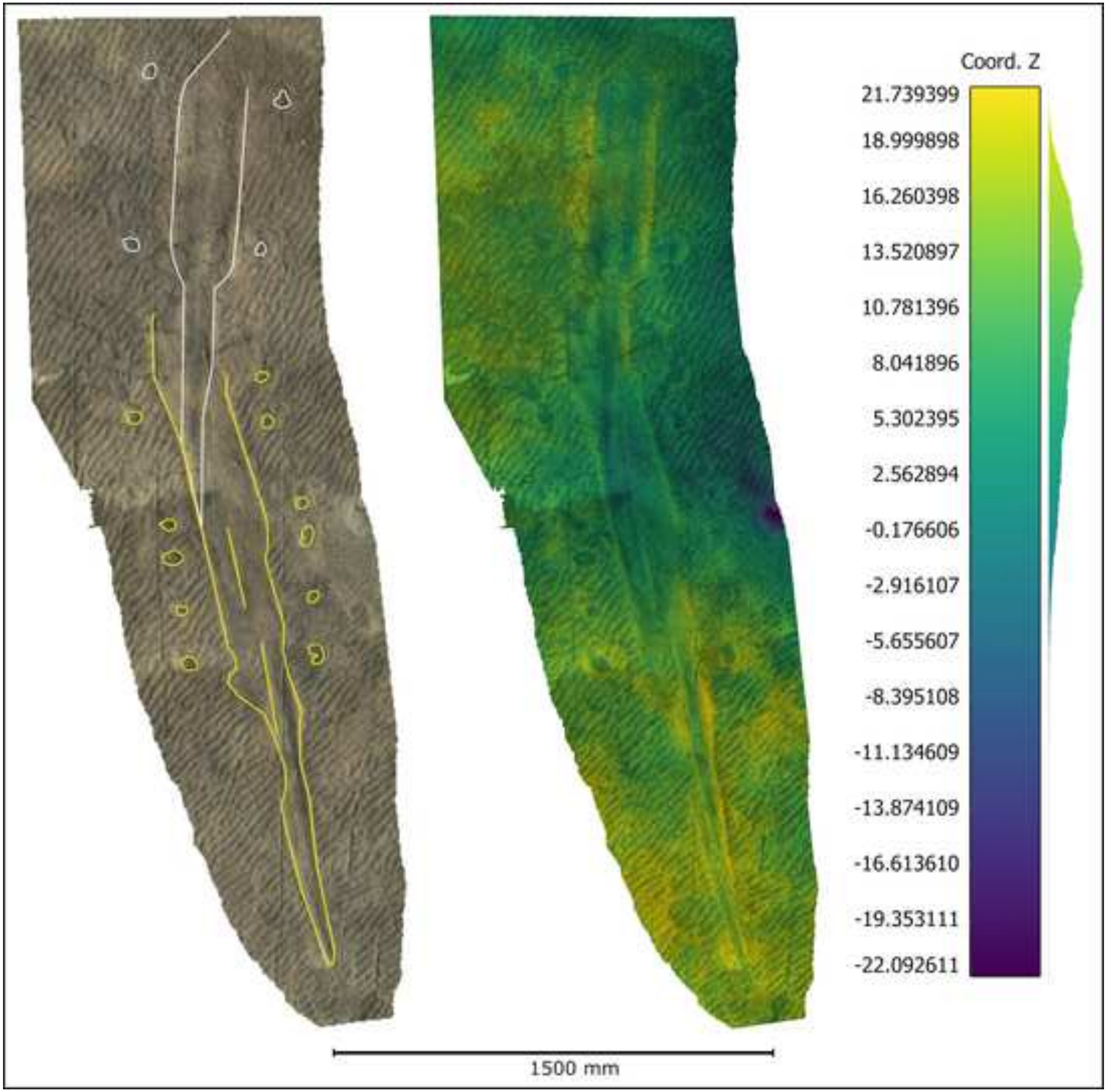

Fig7.tiff

Fig 8

Click here to access/download;Figure;Fig8.tiff

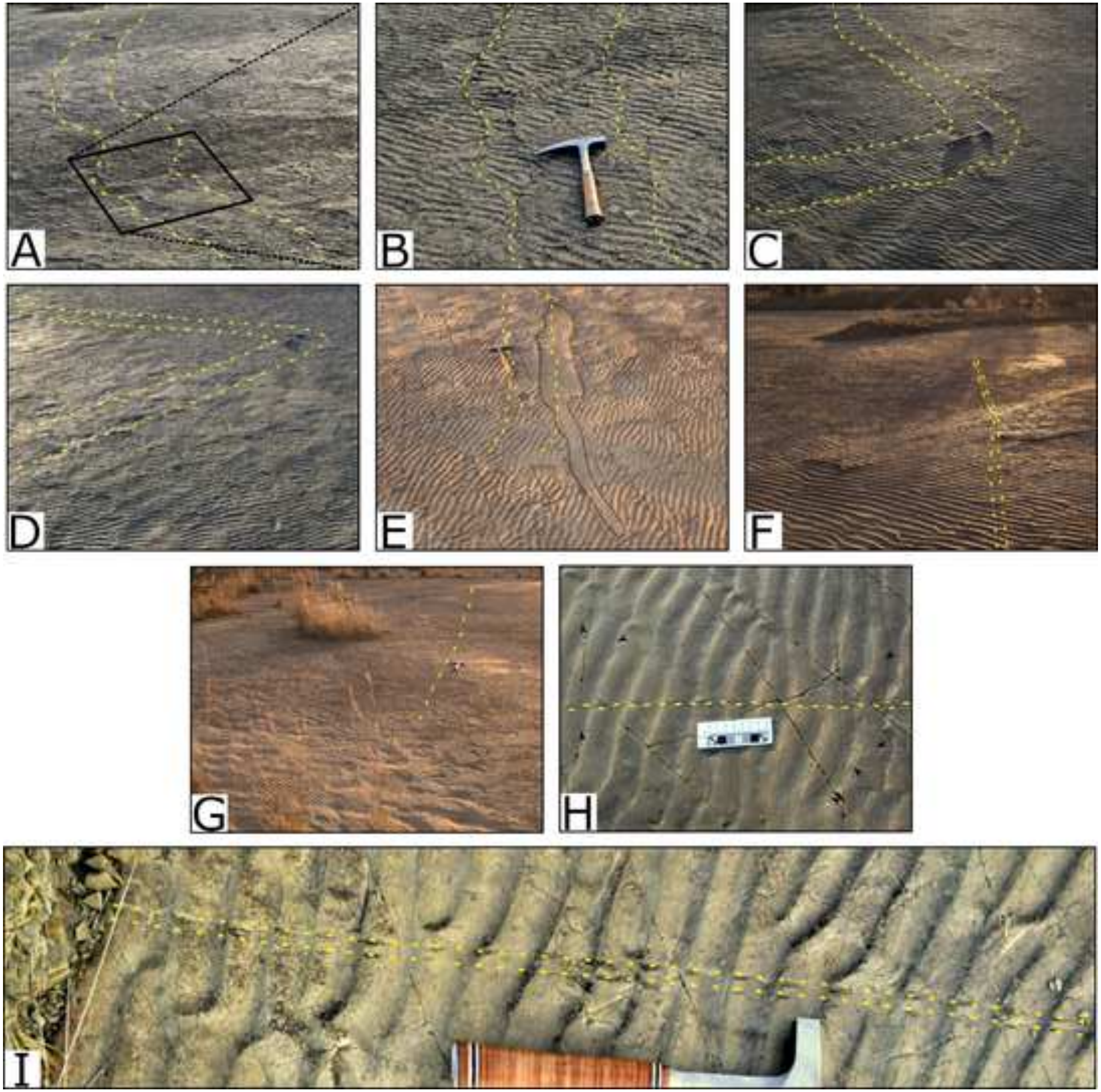

Fig8.tiff

Click here to access/download;Figure;Fig9.tiff

Fig9.tiff

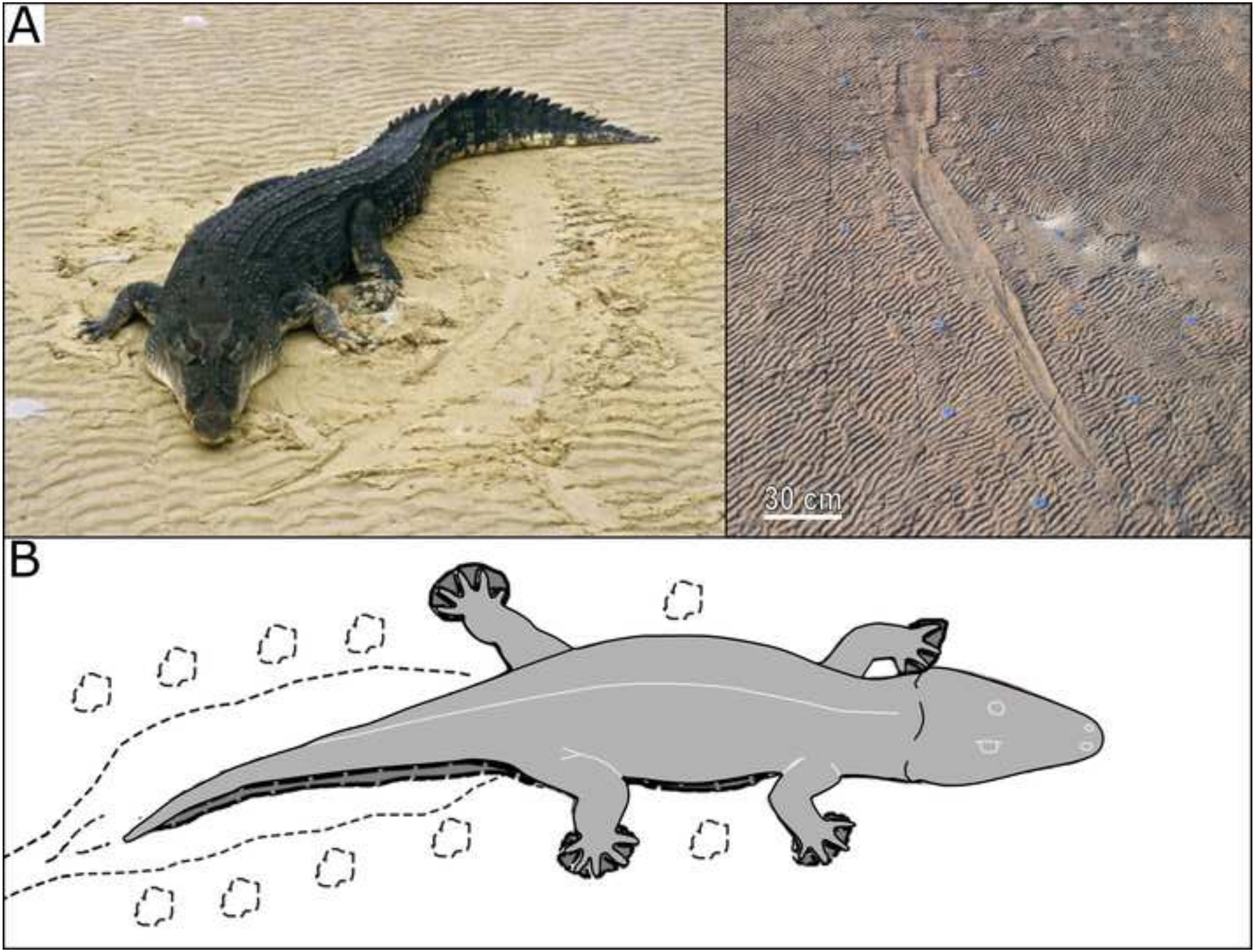

Fig S1

Click here to access/download
Supporting Information
FigS1.tiff

Fig S2

Click here to access/download
Supporting Information
FigS2.tiff

Fig S3

Click here to access/download
Supporting Information
FigS3.tiff
